# Harnessing Disparities in Magnetic Microswarms: From Construction to Collaborative Tasks

**DOI:** 10.1002/advs.202401711

**Published:** 2024-06-13

**Authors:** Chuan Cao, Fangzhi Mou, Manyi Yang, Shuming Zhang, Di Zhang, Luolin Li, Tong Lan, Dunyi Xiao, Wei Luo, Huiru Ma, Jianguo Guan

**Affiliations:** ^1^ State Key Laboratory of Advanced Technology for Materials Synthesis and Processing International School of Materials Science and Engineering Wuhan University of Technology 122 Luoshi Road Wuhan 430070 P. R. China; ^2^ Hubei Key Laboratory of Nanomedicine for Neurodegenerative Diseases Wuhan University of Technology 122 Luoshi Road Wuhan 430070 P. R. China; ^3^ Wuhan Institute of Photochemistry and Technology 7 North Bingang Road Wuhan 430083 P. R. China; ^4^ School of Chemistry Chemical Engineering and Life Science Wuhan University of Technology 122 Luoshi Road Wuhan 430070 P. R. China

**Keywords:** collective behaviors, cooperative functions, heterogeneity, hierarchical organization, micro/nanorobots

## Abstract

Individual differences in size, experience, and task specialization in natural swarms often result in heterogeneity and hierarchy, facilitating efficient and coordinated task accomplishment. Drawing inspiration from this phenomenon, a general strategy is proposed for organizing magnetic micro/nanorobots (MNRs) with apparent differences in size, shape, and properties into cohesive microswarms with tunable heterogeneity, controlled spatial hierarchy, and collaborative tasking capability. In this strategy, disparate magnetic MNRs can be manipulated to show reversible transitions between synchronization and desynchronization by elaborately regulating parameter sets of the rotating magnetic field. Utilizing these transitions, alongside local robust hydrodynamic interactions, diverse heterospecific pairings of disparate magnetic MNRs can be organized into heterogeneous microswarms, and their spatial organization can be dynamically adjusted from egalitarian to leader‐follower‐like hierarchies on the fly, both in open space and complex microchannels. Furthermore, when specializing the disparate MNRs with distinct functions (“division of labor”) such as sensing and drug carrying, they can execute precise drug delivery targeting unknown sites in a collaborative sensing‐navigating‐cargo dropping sequence, demonstrating significant potential for precise tumor treatment. These findings highlight the critical roles of attribute differences and hierarchical organization in designing efficient swarming micro/nanorobots for biomedical applications.

## Graphical abstract

By exploiting synchronization/desynchronization transitions and local robust hydrodynamic interactions, disparate micro/nanorobots with apparent differences in size, shape, and properties, such as chain‐like nanorobots (C‐NRs) and spherical microrobots (S‐MRs), can form cohesive microswarms with tunable heterogeneity and hierarchy. Assigning them with sensing and drug carrying functions enables precise collaborative drug delivery targeting unknown targets through a sensing‐navigating‐cargo dropping sequence.

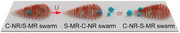

## 1 Introduction

Various organisms within natural ecosystems, ranging from bacterial communities and ant colonies to wolf packs, demonstrate a tendency to form complex collective structures via localized self‐organizing interactions.^[^ ^1^ ^]^ In simple collectives, such as bacterial colonies, individuals share similar morphology, rank, and function, thus exhibiting egalitarian attributes.^[^ ^2^ ^]^ In contrast, social hierarchical collectives, like ant colonies or wolf packs, display notable variations among individuals, primarily manifested in their morphology or group role, and these differences establish a clear linear and fully transmitted hierarchical relationship within the groups.^[^ ^3^ ^]^ Variations among individuals frequently facilitate the division of labor and cooperative dynamics within a group, thereby enabling the successful execution of complex collective activities with enhanced overall efficiency,^[^ ^3b^ ^]^ such as cooperative transportation, migration, foraging, and defense against adversaries.^[^ ^4^ ^]^ From an evolutionary perspective, the hierarchical organization of groups could potentially provide greater efficiency and stability compared to egalitarian structures.^[^ ^3^, ^5^ ^]^ Inspired by these findings, researchers have designed heterogeneous swarm systems of macroscopic robots and formulated their hierarchical control mechanisms.^[^ ^6^ ^]^ These efforts hold considerable potential in advancing the field of intelligent swarm robots.

Micro/nanorobots (MNRs) are artificial tiny machines capable of navigating in many hard‐to‐reach narrow biological environments and executing designated tasks via self‐propulsion or external‐field‐driven propulsion.^[^ ^7^ ^]^ Driven by rapid advances in micro/nanotechnology and inspired by natural self‐organizing systems, research on MNR swarms has recently become a significant focus in the realm of micro/nanorobotics.^[^ ^8^ ^]^ Under various stimuli, including magnetic fields,^[^ ^9^ ^]^ electric fields,^[^ ^10^ ^]^ light,^[^ ^11^ ^]^ and chemical signals,^[^ ^12^ ^]^ etc., many MNRs have demonstrated the ability to self‐organize into cohesive swarms, enabling the accomplishment of complex tasks that cannot be achieved by individual robots alone. Currently, the majority of MNR swarms resemble bacterial colonies, exhibiting characteristics of egalitarian homogeneous swarms consisting of agents that share similar physical and behavioral attributes, restricting their collaborative capabilities.^[^ ^8^ ^]^

To facilitate the execution of sophisticated tasks by MNR swarms, it is probable that introducing heterogeneity—both in attributes and the roles played by individual robots within the collective—is essential.^[^ ^6d^ ^]^ Drawing inspiration from social hierarchical biological colonies, researchers have initiated the integration of MNRs with diverse structures or functionalities to create heterogeneous swarms. Examples include hierarchical swarms that exhibit “leader‐follower‐like” locomotion behavior and active heterogeneous swarms with cooperative self‐propulsion behavior resembling “predator‐prey” dynamics.^[^ ^12^, ^13^ ^]^ Nonetheless, these hierarchical or heterogeneous swarms are constrained by the self‐phoretic propulsion mechanisms of constituents, rendering them easy to fail in biological environments with elevated ionic concentrations and complex compositions. In contrast, magnetically‐powered MNR swarms show great compatibility with biological environments due to their wireless fuel‐free actuation, strong propulsion, and high biocompatibility.^[^ ^14^ ^]^ However, the developed magnetically‐driven heterogeneous swarms consist only of constituent MNRs with minimal differences in size, structure, property, and behavior.^[^ ^15^ ^]^ Despite recent advancements in magnetic MNRs with diverse task specialties,^[^ ^16^ ^]^ organizing these disparate magnetic MNRs into a cohesive heterogeneous swarm poses a considerable challenge since they tend to exhibit different assembly and motion behaviors when subjected to a global alternating magnetic field because of their huge differences in size, composition, structure, and property.

**Figure 1:**
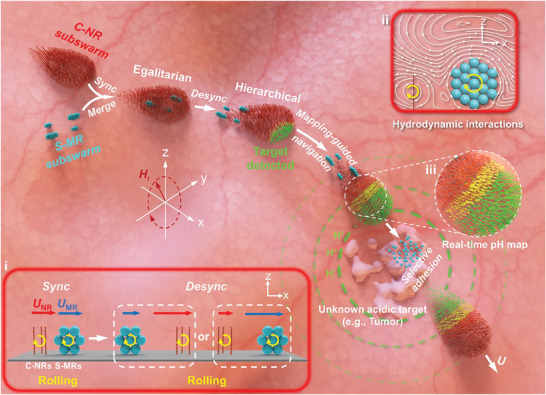
Conceptual design and functions of heterogeneous microswarms comprising C‐NRs and S‐MRs. By elaborately regulating parameter sets of **H**_r_(*t*), disparate magnetic MNRs can be manipulated to show reversible transitions between synchronization (Sync) and desynchronization (Desync) (inset i). Utilizing these transitions, alongside local robust hydrodynamic interactions (inset ii), disparate magnetic MNRs can be organized into heterogeneous microswarms with adjustable group organizations from egalitarian to hierarchical structures. When S‐MRs and C‐NRs are assigned with task specialties of pH sensing and drug carrying, respectively, the heterogeneous swarm in a C‐NRs‐leading organization can perform collaborative precise drug delivery toward unknown targets following a pH mapping (inset iii), mapping‐guided navigation, and carrier dropping sequence.

In this work, we propose a general strategy for organizing disparate magnetic MNRs with significant differences in size, shape, and properties into cohesive microswarms with tunable heterogeneity and controlled spatial hierarchy, and demonstrate their collaborative tasking capability in precise sensing/mapping‐guided drug delivery toward unknown targets (**Figure** **1**). In this strategy, synchronization conditions of disparate magnetic MNRs, such as chain‐like nanorobots (C‐NRs) and spherical microrobots (S‐MRs) (inset i in Figure 1), are at first determined by systematically examining their collective velocities (*U*) in relation to parameter sets of the rotating magnetic field (**H**_r_(*t*)). Under the determined synchronization conditions, the disparate magnetic MNRs, coupled with robust interspecific hydrodynamic interactions (inset ii in Figure 1), can be maneuvered to reach consensus (motion synchronization) and organize into stable heterogeneous swarms. Further, when their synchronization is slightly decoupled, they experience a process akin to “phase segregation” within the group, resulting in the emergence of a leader‐follower‐like hierarchy (inset i in Figure 1). This strategy demonstrates notable control flexibility in group hierarchy and heterogeneity, facilitating the construction of various heterogeneous microswarms with different heterospecific pairings and allowing on‐the‐fly adjustments of their group organizations from egalitarian to leader‐follower‐like hierarchical configurations, both in open space and intricate microchannels. Furthermore, by specializing disparate heterospecific constituents with separate functions (“division of labor”), the heterogeneous swarm can perform complex tasks via sequential task distribution and interspecific collaboration. For instance, when S‐MRs and C‐NRs are respectively allocated with pH sensing and drug carrying functions, the heterogeneous swarm with a C‐NRs‐leading organization can generate a real‐time structural‐color‐based pH map to report the presence and location of an unknown target with abnormal pH levels (inset iii in Figure 1). Then, leveraging the real‐time pH map, the swarm can be intentionally guided to cover the target and deploy the constituent drug‐loaded S‐MRs via selective adhesion, enabling the targeted elimination of tumor cells (Figure 1).

## 2 Results and Discussion

### 2.1 Disparate Robots and Synchronized Swarming Behaviors

**Figure 2:**
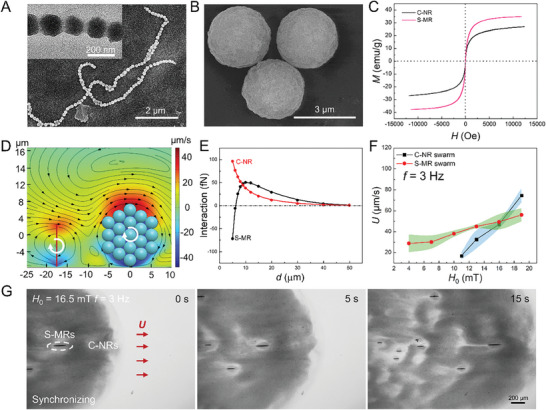
Disparities and synchronized collective motions of C‐NRs and S‐MRs. A,B) SEM images of C‐NRs (A) and S‐MRs (B). The inset in (A) depicts the transmission electron microscopy (TEM) image of a C‐NR. C) Hysteresis loops of C‐NRs and S‐MRs. D) Numerical simulation of the flow field around a wheel‐like S‐MR swarm and a C‐NR. E) Hydrodynamic forces exerted on the C‐NR (red curve, *F*_M‐N_) and the wheel‐like S‐MR subswarm (black curve, *F*_N‐M_) due to interactions with their heterospecific neighbors, which is derived from simulation results in D. F) The *U* of swarming C‐NRs and swarming S‐MRs as a function of *H*_0_ when *f* is kept at 3 Hz. The color ribbons show the varying range of *U* at different *H*_0_. (Sample size, *n* = 5, results are shown as mean ± SD). G) Time‐lapse microscopic images depicting synchronized collective motions of C‐NRs and S‐MRs at a synchronization (*H*_0_, *f*) parameter set of (16.5 mT, 3 Hz). The “dark cloud” is the comet‐like subswarm formed by C‐NRs, and the “large chains” are the standing wheel‐like subswarms formed by S‐MRs.

Generally, magnetic MNRs with task specialties usually display huge differences in size, composition, structure, and property.^[^ ^16^ ^]^ Organizing multiple species of these MNRs into a heterogeneous swarm has the potential to generate intelligent systems with advanced swarm intelligence, complex collaborative multifunctionality, and high task‐accomplishing efficiency. To demonstrate this strategy, the C‐NRs and S‐MRs are at first selected as prototypical instances of two species of disparate magnetic MNRs. As depicted by scanning electron microscopy (SEM) images in **Figure** **2**A,B, the C‐NRs and S‐MRs have different structures and sizes. Specifically, the C‐NRs possess a necklace‐like structure with an average length of 13 µm and a diameter of 150 nm (Figure 2A), in which the Fe_3_O_4_ nanoparticles were 1D arranged and enveloped in a thin hydrogel shell (the inset in Figure 2A). In contrast, the S‐MRs have a spherical morphology with a diameter of 3 µm (Figure 2B). Furthermore, it is worth noting that the C‐NRs and S‐MRs display distinct magnetic properties. As evidenced by their respective hysteresis loops, the S‐MRs exhibit a high saturated mass magnetization of 34.8 emu g^−1^ at room temperature, which is approximately 30% greater than that of the C‐NRs (26.8 emu g^−1^) (Figure 2C). Due to significant differences in size, shape, and magnetic properties, the C‐NRs and S‐MRs usually show different collective structures and mobilities under the same rotating **H**_r_(*t*). For instance, when driven by a rotating **H**_r_(*t*) with a strength (*H*_0_) of 16 mT and a frequency (*f*) of 7 Hz, those C‐NRs form into comet‐like swarms primarily via hydrodynamic coupling (Figure S1A and Video S1, Supporting Information), while the S‐MRs self‐organize with their neighbors into wheel‐like swarms mainly through local magnetic interactions (Figure S1B and Video S1, Supporting Information). In addition, the comet‐like C‐NR swarms show an average *U* of 15 µm s^−1^, which is notably lower than that observed in the wheel‐like S‐MR swarms (80 µm s^−1^), as also verified by different displacements of their swarm front in the same period time of 15 s (Figure S1A,B, Supporting Information). Rolling MNRs are known to generate strong vortices, exerting long‐range hydrodynamic attraction on their neighbors.^[^ ^17^ ^]^ Thus, these interactions may bridge the gap in collective mobilities between C‐NRs and S‐MRs, facilitating their interspecific aggregation. To visualize the interspecific hydrodynamic interaction, the flow field around a wheel‐like S‐MR swarm and a C‐NR was simulated. As illustrated in Figure 2D, a clockwise‐rolling wheel‐like S‐MR swarm impels the fluid rightward with a velocity *u* in the *x* direction (the translational direction of the S‐MR swarm), thereby creating a femtonewton‐scale hydrodynamic pull (or attraction, *F*_M‐N_) on the nearby C‐NR. This *F*_M‐N_ increases as the distance (*d*) between the S‐MR swarm and the C‐NR decreases (Figure 2E), suggesting that nearby C‐NRs are dragged and accelerated toward the S‐MR swarm to facilitate their aggregation with the S‐MR swarm. Likewise, a rolling C‐NR also exerts a hydrodynamic force (*F*_N‐M_) on the S‐MR swarm (Figure 2E). This force demonstrates an attractive nature when the S‐MR swarm and the rolling C‐NR are in close proximity (*d* ≤ 6.2 µm in the simulated case, Figure 2E), facilitating their aggregation and synchronization. On the other hand, it transforms into a hydrodynamic push (or repulsion) when the rolling C‐NR is distant from the S‐MR swarm (*d* > 6.2 µm). Consequently, it is rational to speculate that nearby rolling C‐NRs tend to aggregate with the S‐MR swarm, while those farther away exert a collective hydrodynamic push to it.^[^ ^9c^ ^]^ These findings confirm that the hydrodynamic interactions not only enable interspecific aggregation but also facilitate mutual boosting of disparate S‐MRs and C‐NRs.

Through a systematic investigation of the *U* dependency of both C‐NRs and S‐MRs on **H**_r_(*t*) parameters, it becomes straightforward to identify synchronous conditions between them. Specifically, when a fixed *f* value (3 Hz) is considered, the swarming S‐MRs demonstrate a gradual increase in velocity as *H*_0_ varies from 4 to 19 mT, while the swarming C‐NRs display a sudden surge in velocity as *H*_0_ increases, attributed to the pronounced hydrodynamic coupling among crowded C‐NRs (Figure 2F). Consequently, the *H*_0_‐dependent *U* curves of the swarming C‐NRs and S‐MRs intersect at a (*H*_0_, *f*) set of (16.5 mT, 3 Hz), indicating the condition where they exhibit a similar collective velocity (Figure 2F). When the C‐NRs and S‐MRs were mixed and driven by a rotating **H**_r_(*t*) with the optimized (*H*_0_, *f*) set of (16.5 mT, 3 Hz), they, coupled with interspecific hydrodynamic interactions, could collectively move forward as a cohesive heterogeneous swarm (Figure 2G; Video S3, Supporting Information). Within the heterogeneous swarm, the C‐NRs and S‐MRs can be clearly distinguished, and their relative positions exhibit negligible changes during the collective motion, revealing their motion synchronization in the heterogeneous swarm (Figure 2G). The side‐view observation reveals that the entire swarm has a continuous comet‐like structure with a denser head and a thinner tail, and no obvious group disassociation is observed (Figure S2 and Video S2, Supporting Information). Using similar processes, synchronization conditions between the swarming C‐NRs and S‐MRs were also observed at (*H*_0_, *f*) sets of (8.5 mT, 1 Hz) and (18.5 mT, 5 Hz), respectively, and thus they could form into heterogeneous swarms at these (*H*_0_, *f*) sets as well (Figure S3A,B and Video S3, Supporting Information). To further confirm this synchronicity, we calculated the instantaneous speed of the C‐NR subswarm and S‐MR subswarms within the heterogeneous swarm under the three optimized (*H*_0_, *f*) parameter sets (Figure S3C–E, Supporting Information). The results indicated that even though both the C‐NR subswarm and S‐MR subswarms exhibited notable fluctuations in their instantaneous speeds over time, an enduring overall speed consistency was observed. In this context, the formed heterogeneous swarm exhibits an egalitarian group organization, reminiscent of an ant colony during a foraging expedition, where both soldier ants and worker ants, despite their differing sizes and roles, march at comparable speeds to optimize food collection.

Furthermore, we have investigated the influence of the mass ratio of C‐NRs to S‐MRs (*m*_C‐NRs_:*m*_S‐MRs_), fluid viscosity, and surface conditions on the formation and collective motion of the heterogeneous swarms. When the ratio was adjusted from 24:3 to 8:9, it was observed that under the (*H*_0_, *f*) parameter set of (16.5 mT, 3 Hz), the S‐MR subswarms and the C‐NR subswarm moved synchronously with a similar *U* of 62 µm s^−1^ (Figure S4 and Video S4, Supporting Information). However, they tended to desynchronize when the ratio fell outside this range (Figure S4 and Video S4, Supporting Information). Specifically, at ratios of 56:3 and 40:3, the C‐NRs aggregated into a large and energetic C‐NR subswarm, exhibiting a much higher *U* (≈ 46 µm s^−1^) than the scattered small S‐MR subswarms (≈ 20 µm s^−1^). Conversely, when the ratio was decreased to 8:15 and 8:21, large S‐MR subswarms formed and displayed a much higher *U* (≥ 78 µm s^−1^) than the thin C‐NR subswarm (≈ 60 µm s^−1^). Additionally, some slow‐moving C‐NRs could be trapped by the flow field of the S‐MR subswarm and moved along with it as puppet followers. Furthermore, synchronization of C‐NRs and S‐MRs has been observed at (*H*_0_, *f*) parameter sets of (11.8 mT, 1 Hz) and (14 mT, 1 Hz) in the medium with viscosities of 1.3 and 3.0 cP, respectively (see Figure S5A,B, Supporting Information). When C‐NRs and S‐MRs were mixed and driven by the determined synchronization **H**_r_(*t*) parameter sets, they could collectively move forward as a cohesive heterogeneous swarm in the viscous media (see Figure S5C and Video S5, Supporting Information). Finally, we investigated the influence of different surface conditions on the collective behaviors of the heterogeneous swarms (Figure S6). As illustrated in Figure S6B,C (Supporting Information), on both the plastic substrate and the glass slide seeded with endothelial cells, C‐NRs and S‐MRs were still able to achieve synchronized motion at the (*H*_0_, *f*) parameter set of (8.5 mT, 1 Hz), forming egalitarian heterogeneous swarms similar to those on the glass substrate (Figure S6A, Supporting Information). Within the same duration of motion (10 s), the displacement generated by the heterogeneous swarms on the plastic and endothelial cell substrates was almost identical to that on the glass substrate, with a movement velocity of about 20 µm s^−1^ (Video S6, Supporting Information).

### 2.2 Hierarchical Control over Heterogeneous Swarms

**Figure 3:**
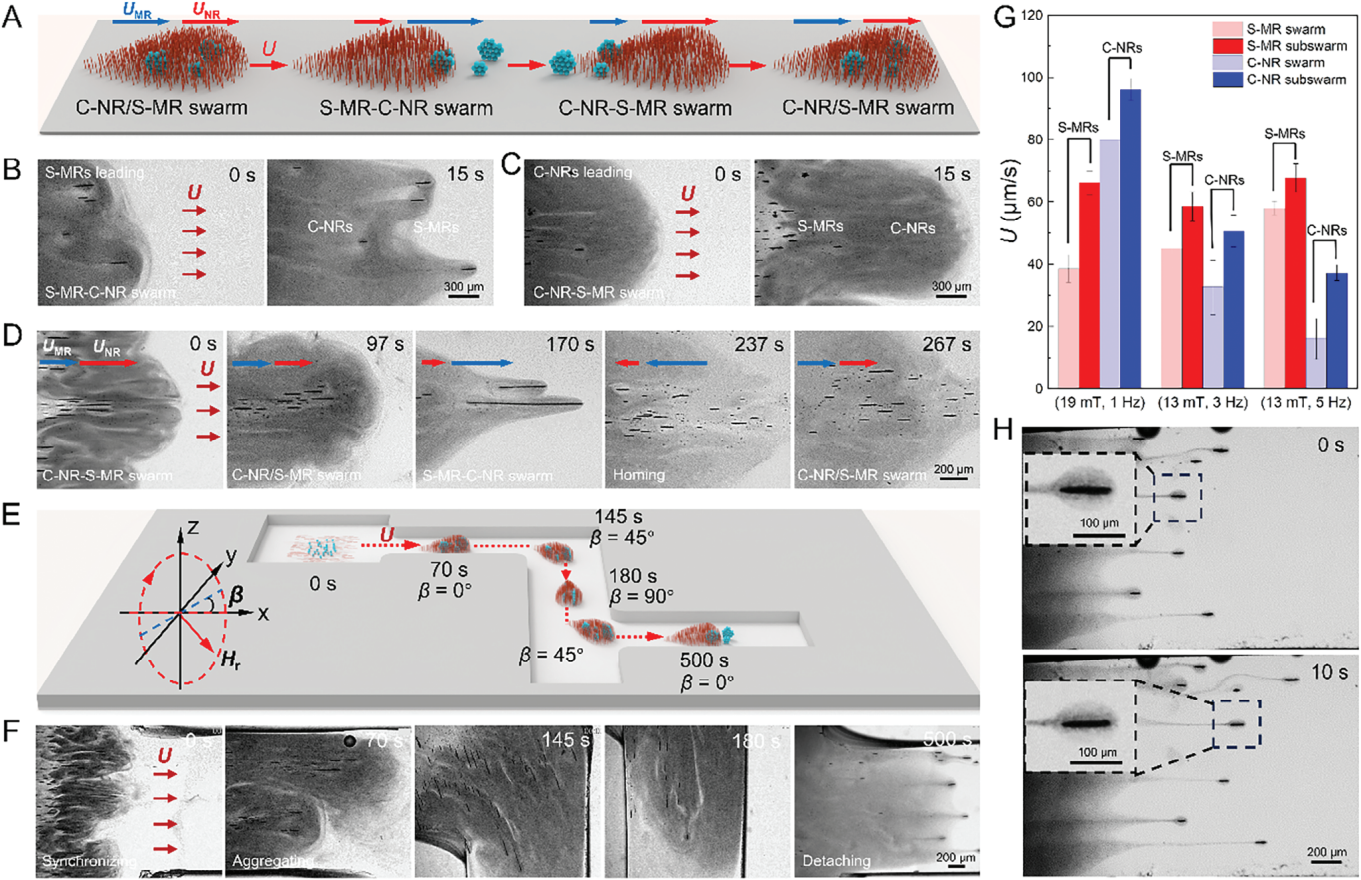
Hierarchical control over the heterogeneous swarms. A) Schematic diagram illustrating group configuration transformations of the heterogeneous swarm, facilitating its reversible transitions from an egalitarian C‐NR/S‐MR swarm to hierarchical S‐MR‐C‐NR or C‐NR‐S‐MR leader‐follower‐like swarms. *U*_NR_ and *U*_MR_ are the collective velocities of C‐NRs and S‐MRs, respectively. B, C) Time‐lapse microscopic images depicting collective motions of hierarchical S‐MR‐C‐NR (B) and C‐NR‐S‐MR (C) heterogeneous swarms. D) Dynamic group configuration transformations of a heterogeneous swarm in open space. E, F) Schematic diagram (E) and time‐lapse microscopic images (F) depicting a heterogeneous swarm transversing a complex microchannel with multiple sharp turns by dynamically adjusting (*H*_0_, *f*) parameter sets and orientation angle (*β*) of the rotating **H**_r_(*t*). G) The *U* of homogeneous C‐NR and S‐MR swarms in contrast to the C‐NR and S‐MR subswarms in the heterogeneous swarms at different (*H*_0_, *f*) parameter sets. (*n* = 5, results are shown as mean ± SD). H) Time‐lapse microscopic images showing the collective motion of the trapped C‐NRs alongside wheel‐like S‐MR subswarms when driven at an (*H*_0_, *f*) parameter set of (8.5 mT, 3 Hz).

When the **H**_r_(*t*) parameters were adjusted to slightly deviate from the synchronization conditions, the C‐NRs and S‐MRs can be navigated to move at different *U* (Figure 2F; Figure S3A,B, Supporting Information). This adjustment would lead to the emergence of leaders and followers within the swarm, ultimately transforming the originally egalitarian heterogeneous swarm into a hierarchical heterogeneous swarm, such as the S‐MR‐C‐NR or C‐NR‐S‐MR leader‐follower‐like swarm (**Figure** **3**A). For instance, at a (*H*_0_, *f*) parameter set of (11 mT, 3 Hz), as the velocity of the S‐MR subswarms (*U*_MR_) is greater than that of the C‐NR subswarm (*U*_NR_) (*U*_MR_ > *U*_NR_) (Figure S7A, Supporting Information), the S‐MR subswarms gradually concentrated at the front of the heterogeneous swarm, resulting in a S‐MR‐C‐NR leader‐follower‐like swarm (Figure 3B; Video S7, Supporting Information). When the (*H*_0_, *f*) parameter set of the rotating **H**_r_(*t*) was set at a value of (16 mT, 1 Hz), the C‐NR subswarm gradually emerged as the leader as *U*_NR_ > *U*_MR_ (Figure S7B, Supporting Information), thus realizing the construction of a C‐NR‐S‐MR leader‐follower‐like heterogeneous swarm (Figure 3C; Video S7, Supporting Information). Afterward, the rotating **H**_r_(*t*) parameter was readjusted to the synchronization condition, allowing the heterogeneous swarm to maintain its predetermined group configuration during its subsequent collective motion. These results suggest that when the disparate heterospecific constituents are slightly desynchronized, the heterogeneous swarm undergoes a process resembling “phase segregation”, and thus can easily switch its group organization from an egalitarian to a hierarchical configuration.

Further, as the synchronization of heterogeneous constituents can be established and disrupted by manipulating the (*H*_0_, *f*) parameters of the rotating **H**_r_(*t*), dynamic hierarchical control over the heterogeneous swarm is readily achievable on the fly (Figure S7C, Supporting Information). The experimental results are given in Figure 3D and Video S8 (Supporting Information). Initially, under a rotating **H**_r_(*t*) with a (*H*_0_, *f*) parameter set of (9 mT, 1 Hz), the C‐NRs and S‐MRs self‐organized into a hierarchical heterogeneous C‐NR‐S‐MR swarm, with the C‐NR subswarm leading the group (0 s in Figure 3D). Subsequently, after the S‐MR subswarm was accelerated to catch up with the C‐NR subswarm at a (*H*_0_, *f*) parameter set to (8.5 mT, 3 Hz), both subswarms were encouraged to exhibit comparable *U* by adjusting the (*H*_0_, *f*) parameter set to a synchronization (*H*_0_, *f*) parameter set of (8.5 mT, 1 Hz), resulting in the formation of an egalitarian heterogeneous C‐NR/S‐MR swarm (97 s in Figure 3D). As the *f* was increased to 3 Hz while maintaining *H*_0_ unchanged, the two subswarms once again exhibited speed discrepancies, leading to the emergence of a S‐MRs‐leading swarm structure (i.e., S‐MR‐C‐NR swarm) over time (170 s in Figure 3D). Afterward, while keeping the (*H*_0_, *f*) parameter constant and reversing the orientation of **H**_r_(*t*), the motion direction of the swarm was reversed. Owing to the difference in *U* between the subswarms, the S‐MR subswarms converged back to the C‐NR subswarm, a phenomenon referred to as the homing of S‐MRs (237 s in Figure 3D). Finally, the orientation of **H**_r_(*t*) was reversed once again, and the (*H*_0_, *f*) parameter was adjusted to (8.5 mT, 1 Hz), prompting both subswarms to synchronize their motion rightward, thereby reestablishing the egalitarian C‐NR/S‐MR swarm structure (267 s in Figure 3D). The above experimental results demonstrate the dynamic controllability of the heterogeneous swarm in group configurations.

Leveraging precise directional control, the heterogeneous swarm can navigate through complex microchannels in controlled group configurations (Figure 3E; Video S9, Supporting Information). When the mixture of C‐NRs and S‐MRs at the channel entrance was actuated by a rotating **H**_r_(*t*) with a (*H*_0_, *f*) parameter set of (8.5 mT, 1 Hz), they collectively moved rightward and funneled into the entrance (0 s in Figure 3F). During this stage, due to the rapid increase in the local number density of C‐NRs resulting from the confining effect of the microchannel entrance, the C‐NR subswarm moved slightly faster. To facilitate prompt aggregation and synchronization of the two subswarms, the *f* of the **H**_r_(*t*) was increased to 3 Hz, thereby boosting the *U* of the S‐MR subswarms. After a certain duration, the S‐MR subswarms caught up with the C‐NR subswarm, and then, by resetting the *f* to 1 Hz, they achieved motion synchronization and subsequently cruised in an egalitarian organization thereafter (70 s in Figure 3F). When approaching sharp turns, the orientation of the rotating **H**_r_(*t*), denoted by the angle *β* between the rotating plane and the *x*‐*z* plane, was adjusted incrementally by 5° at each step to ensure the cohesiveness of the swarm structure until the entire swarm completed the turn. In this way, the heterogeneous swarm smoothly traversed the microchannel with two sharp turns (145‐500 s in Figure 3F). Upon approaching the destination (the right end of the microchannel), the S‐MR subswarms could be strategically detached from the heterogeneous swarm by accelerating them using a (*H*_0_, *f*) parameter set of (8.5 mT, 3 Hz), enabling them to reach the destination ahead of the rest of the swarm (500 s in Figure 3F).

To investigate the effects of hydrodynamic interactions on synchronization and collective motions of disparate S‐MRs and C‐NRs during hierarchical control (or “phase segregation”), we examined the *U* of the S‐MR and C‐NR subswarms within the heterogeneous swarm and compared it to that of homogeneous swarms composed solely of S‐MRs or C‐NRs. As anticipated by the numerical simulation results in Figure 2D,E, the S‐MRs and C‐NRs within the heterogeneous swarms consistently exhibited higher *U* than their counterparts in the homogeneous swarms at varying (*H*_0_, *f*) parameter sets, verifying the mutual boosting effect from the interspecific hydrodynamic interactions (Figure 3G). This mutual boosting effect in the heterogeneous swarm becomes particularly pronounced when there is a significant discrepancy in the velocities of swarming S‐MRs and C‐NRs (Figure 3G). In addition, in comparison to their counterparts in the homogeneous swarms at the same (*H*_0_, *f*) parameter sets of (13 mT, 3 Hz), (19 mT, 1 Hz), and (13 mT, 5 Hz), slower constituents (C‐NRs or S‐MRs) within the heterogeneous swarm experienced substantial boosts of 55.7%, 72.2%, and 131.0%, while the faster constituents exhibited slight boosts of only 30.0%, 20.5%, and 17.1%, respectively (Figure 3G). This uneven boosting effect significantly reduces the velocity differences (Δ*U*, Δ*U* = *U*_NR_ − *U*_MR_) between S‐MRs and C‐NRs within the heterogeneous swarms compared to their counterparts in homogeneous swarms, further confirming that interspecific hydrodynamic interactions can partially facilitate the synchronization of disparate constituents. Using passive tracers of polystyrene (PS) microspheres (2 µm), the flow fields around swarming C‐NRs and S‐MRs can be visualized (Figure S8 and Video S10, Supporting Information). The results reveal that, during the collective motion, the swarming C‐NRs generate a large recirculatory flow field that may advect the S‐MR subswarms (Figure S8A and Video S10, Supporting Information), while the S‐MR subswarms generate small standing vortices nested within the global recirculatory flow, attracting and energizing nearby C‐NRs (Figure S8B and Video S10, Supporting Information). Similarly, in some extreme circumstances, slow C‐NRs became entrapped by the strong flow field generated by ultrafast wheel‐like S‐MR swarms nearby that detached from the heterogeneous swarm, enabling them to move alongside it as slave followers at the same *U* (Figure 3H).

### 2.3 Heterogeneous Swarms of Different Heterospecific Pairings

**Figure 4:**
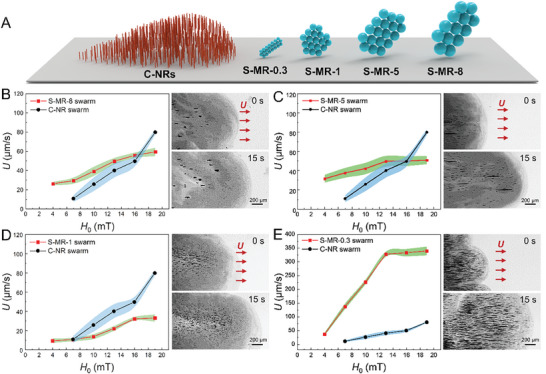
Heterogeneous swarms of different heterospecific pairings. A) Schematic illustration of the C‐NRs and the S‐MRs with different sizes (denoted as S‐MR‐*a*, respectively, where *a* is in a unit of µm) used for the construction of heterogeneous swarms. B–E) Collective velocity *U* of C‐NR swarms as a function of *H*_0_ in contrast to that of the S‐MR‐8 (B), S‐MR‐5 (C), S‐MR‐1 (D), and S‐MR‐0.3 (E) swarms (*n* = 5, results are shown as mean ± SD) (left panel), and time‐lapse microscopic images (right panels) depicting collective motion of the corresponding heterogeneous swarms driven at the determined synchronization *H*_0_ parameters. The *f* in B‐E is kept at 1 Hz.

Given the robust interspecific hydrodynamic interactions and easy identification of synchronization conditions, the proposed strategy holds significant potential for constructing a wide range of heterogeneous microswarms comprising various species of magnetic MNRs. To demonstrate its versatility, we first constructed different C‐NR/S‐MR heterogeneous swarms by varying the size of the S‐MRs from 1 to 8 µm (**Figure** **4**A; Figure S9A–C, Supporting Information). Specifically, by scanning the *U* of the different‐sized S‐MRs at different *H*_0_ and a fixed *f* of 1 Hz and comparing it to that of the C‐NRs, the synchronization conditions between the C‐NRs and the S‐MRs with a size of 8, 5, and 1 µm were identified to be at a *H*_0_ parameter of 16.5, 16, and 7 mT, respectively (left panel in Figure 4B–D). Once the synchronization conditions were determined, the C‐NRs were mixed with the S‐MRs, and subsequently propelled using the corresponding **H**_r_(*t*) parameters (*H*_0_). As shown in the time‐lapse optical microscopic images of Figure 4B‐D and Video S11 (Supporting Information), the C‐NRs can organize with the S‐MRs into heterogeneous swarms regardless of different sizes of S‐MRs under the identified synchronization **H**_r_(*t*) parameters. Notably, when the size of S‐MRs was reduced to 0.3 µm (Figure S9D, Supporting Information), a dimension close to that of the C‐NRs (0.15 µm), despite a significant discrepancy in *U*, they were still able to synchronize and form a stable heterogeneous swarm with the C‐NRs (Figure 4E; Video S11, Supporting Information). This result suggests that synchronized swarming motion becomes readily achievable among heterospecific constituents within the group when they share similar sizes owing to robust local interspecific hydrodynamic interactions. On the other hand, this strategy is also feasible to build stable heterogeneous swarms when the C‐NR constituents are replaced with spherical magnetic nanoparticles (150 nm in size, Figure S9E, Supporting Information) utilizing similar synchronization condition identification procedures (Figure S10 and Video S11, Supporting Information). These results reveal that the proposed strategy can be used to construct heterogeneous swarms with tunable heterogeneity, including varying levels of heterogeneity (e.g., size differences) and diverse heterospecific pairings.

### 2.4 Collaborative Precise Drug Delivery

**Figure 5:**
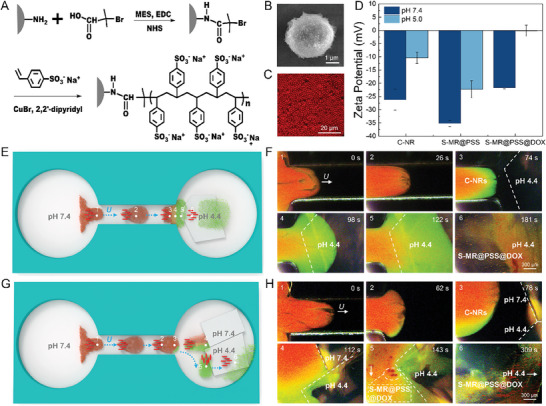
Collaborative precise drug delivery by the heterogeneous swarm. A) Schematic demonstration of the preparation of S‐MRs grafted with polystyrene sulfonate sodium (PSS) brushes (S‐MR@PSS). B) The SEM image of a typical S‐MR@PSS particle. C) The fluorescent microscopic image of the S‐MR@PSS particles loaded with DOX (S‐MR@PSS@DOX particles). D) Zeta potential of C‐NRs, S‐MR@PSS particles, and S‐MR@PSS@DOX particles at pH 7.4 and 5.0, respectively (*n* = 3, results are shown as mean ± SD). E–H) Schematic illustration (E, G) and time‐lapse dark‐field microscopic images (F, H) of a C‐NR‐S‐MR@PSS@DOX heterogeneous swarm performing precise drug delivery when collectively moving from a reservoir (pH 7.4) toward an unknown low‐pH target (a pH 4.4 agar gel) without (E, F) or with an interference (a pH 7.4 agar gel) (G, H).

By assigning distinct roles (a form of “labor division”) to C‐NRs and S‐MRs, we unlock a collaborative synergy that empowers them to tackle complex tasks. For instance, when the C‐NRs and S‐MRs are respectively designated for the tasks of environmental sensing and drug carrying, it gives rise to the formation of heterogeneous sensor‐carrier swarms, poised to execute precise drug delivery toward hitherto unknown targets. It is noteworthy that the C‐NRs used in this work exhibit an inherent capacity to function as pH nanosensors, characterized by their ability to exhibit distinct structural colors upon variations in pH levels. Specifically, when the pH value in the surrounding environment increases, carboxyl groups in the poly(AA‐co‐HEA) hydrogel scaffold of the C‐NRs deprotonate into carboxylates, resulting in the swelling of the hydrogel scaffold due to the higher solubility of the latter than the former. As the monodispersed Fe_3_O_4_ nanoparticles are fixed in the hydrogel scaffold, the swelling of the hydrogel scaffold makes the interparticle distance *d*, namely, the lattice constant of the encapsulated 1D periodic assemblies, become larger, leading to the redshift of the diffracted color (or diffraction peak) of the C‐NRs according to Bragg's law.^[^ ^18^ ^]^ On the other hand, when the surrounding pH value decreases, carboxylates in the poly(AA‐co‐HEA) hydrogel scaffold protonate to carboxyl groups, leading to hydrogel shrinking and the blue shift of the diffracted color of the C‐NRs. This unique attribute endows C‐NRs with the capability to monitor and visualize localized deviations in pH, thereby facilitating the identification of uncharted targets on the fly. To endow the S‐MRs with drug‐carrying functions, they were elaborately grafted with polystyrene sulfonate sodium (PSS) brushes using a surface‐initiated atom transfer radical polymerization (ATRP) method (**Figure** **5**A, see detail in Experimental Section).^[^ ^19^ ^]^ The PSS grafted S‐MRs (S‐MR@PSS) exhibited an obvious increase in hydrodynamic size from 3.3 to 3.7 µm as a function of ATRP time (*t*), indicating the formation of a polymer shell with thicknesses ranging from 220 to 620 nm enveloping the surface of the unmodified S‐MRs (3.0 µm), respectively (Figure S11A, Supporting Information). The successful grafting of PSS brushes was further confirmed by SEM observation and Fourier transforms infrared (FT‐IR) analysis of the S‐MR@PSS particles that were obtained at a *t* of 9 h. The SEM image in Figure 5B reveals that the microrobot has a notably rougher surface after grafting PSS brushes in contrast to the pristine S‐MR (Figure S9F, Supporting Information). In the FT‐IR spectrum presented in Figure S11B (Supporting Information), characteristic bands of surface PSS brushes are evident, including the stretching vibration of the benzene ring skeleton at 1449 cm^−^¹ and the stretching vibrations of ‐SO_3_ at 1055 and 1023 cm^−^¹.

The pH sensitivity of C‐NRs was assessed by tracking alterations in their structural colors with varying pH values. As the pH of the aqueous medium increased from 4.0 to 7.4, the C‐NRs displayed a broad spectrum of structural color shifts, transitioning from dark blue to bright red (Figure S12, Supporting Information). The drug loading on S‐MR@PSS particles was achieved by immersing them directly in a solution of doxorubicin (DOX) hydrochloride, thereby facilitating DOX adsorption onto the particles through electrostatic interactions.^[^ ^20^ ^]^ As confirmed by the fluorescence microscopic image in Figure 5C, it is evident that DOX molecules were effectively loaded onto the S‐MR@PSS particles, with the loading capacity quantified at 26 wt.%. Notably, DOX‐loaded S‐MR@PSS (S‐MR@PSS@DOX) particles also exhibit a pH‐responsive behavior in their zeta potential (*ζ*). As the pH level decreased from 7.4 to 5.0, the *ζ* of the S‐MR@PSS@DOX particles underwent a transition from −21.6 mV to nearly 0 (Figure 5D). In contrast, C‐NRs and S‐MR@PSS maintained their negative *ζ* values even as the pH of the surrounding medium decreased to 5.0 (Figure 5D). This pH sensitivity may potentially enhance the targeted drug delivery of S‐MR@PSS@DOX particles through selective adhesion on the targets with abnormal pH levels.^[^ ^21^ ^]^

Equipped with the designated functionalities, the C‐NRs and S‐MR@PSSs within a heterogeneous swarm can execute precise drug delivery targeting uncharted targets via interspecific collaborations. In this drug delivery mission, the establishment of a leader‐follower‐like group configuration is of paramount importance, as the initial identification and visualization of uncharted targets represent a critical initial phase in planning the precise drug delivery route. To realize the requisite group configuration control, conditions for synchronization and desynchronization (or phase segregation) of C‐NRs and S‐MR@PSS@DOX particles were investigated both in open space and within a microchannel (Figure S13A,B, Supporting Information). Subsequently, an optimized (*H*_0_, *f*) parameter set of (16 mT, 1 Hz) was selected to power the heterogeneous swarm, ensuring the establishment of a C‐NR‐S‐MR@PSS@DOX leader‐follower‐like group configuration.

The investigation of precise drug delivery was conducted within a microfluidic chip featuring two open reservoirs interconnected by a narrow canal. The C‐NR‐S‐MR@PSS@DOX swarm was driven by the **H**_r_(*t*) with the predetermined (*H*_0_, *f*) set, directing it toward the right reservoir housing a piece of pH 4.4 agar gel serving as the designated target (Figure 5E). Before the swarm reached the right reservoir, the C‐NRs within the heterogeneous swarm exhibited a bright red structural color, representing the pH of the surrounding aqueous medium (pH 7.4 buffer) (0‐26 s in Figure 5F; Video S12, Supporting Information). Upon nearing the designated agar gel target by ≈ 500 µm (74 s in Figure 5F; Video S12, Supporting Information), the C‐NRs at the leading edge of the swarm changed their structural color from red to green, signifying the detection of the low‐pH target. As the swarm covered the gel target and its immediate vicinity, a discernible structural‐color pH map promptly emerged (98‐122 s in Figure 5F; Video S12, Supporting Information). Then, the rolling negatively charged C‐NRs (*ζ* = −10 mV at pH 4.4) at the forefront gradually distanced themselves from the gel target, utilizing their robust repulsion with the gel surface based on hydrodynamic and electrostatic interactions (Figure S14, Supporting Information). Meanwhile, the S‐MR@PSS@DOX particles at the rear of the swarm selectively adhered to the low‐pH gel surface, owing to their negligible surface charge (*ζ* ≈ 0 mV at pH 4.4) once they reached the low‐pH target (181 s in Figure 5F; Video S12, Supporting Information).

Furthermore, the C‐NR‐S‐MR@PSS@DOX swarm can distinguish a legitimate target from potential interference, thereby effectively guiding itself to the intended location. In this experiment, two agar gels were placed in the right reservoir, one serving as the target with a pH of 4.4, and the other as interference with a pH of 7.4 (Figure 5G). As the C‐NR‐S‐MR@PSS@DOX swarm approached the agar gels, only the C‐NRs located at the lower right front of the swarm emitted a green signal, indicating the presence of the low‐pH target in that particular direction (0‐78 s in Figure 5H; Video S13, Supporting Information). In response to this feedback, the swarm was intentionally steered to execute a right and then a left turn to cover the low‐pH target, facilitating the precise delivery of S‐MR@PSS@DOX particles to the target area (143‐309 s in Figure 5H; Video S13, Supporting Information). These findings highlight the capability of the swarm to effectuate precise drug delivery in a sequential process through the collaborative efforts of the sensing C‐NRs and the drug‐loaded S‐MR@PSS@DOX particles.

### 2.5 Targeted Elimination of Tumor Cells

**Figure 6:**
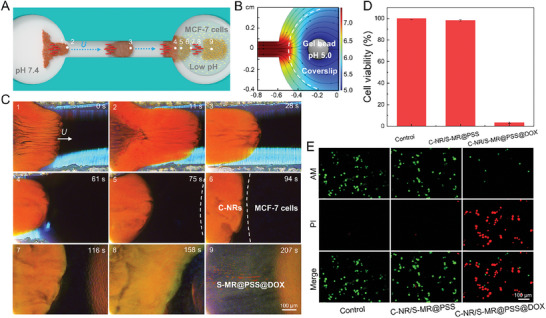
Targeted tumor‐cell elimination by the heterogeneous swarm. A) Schematic diagram depicting the mapping‐guided drug delivery toward MCF‐7 tumor cells by a C‐NR‐S‐MR@PSS@DOX swarm. B) Numerical simulation of pH distribution around MCF‐7 cells on a coverslip with a pH 5.0 gel bead placed on it. C) Time‐lapse dark‐field microscopic images depicting the detection of MCF‐7 cells in the low‐pH region and the subsequent guided delivery of S‐MR@PSS@DOX particles. D) MCF‐7 cell viability before (Control) and after incubation with C‐NR/S‐MR@PSS and C‐NR/S‐MR@PSS@DOX mixtures for 4 h, respectively. (*n* = 3, results are shown as mean ± SD). E) Live/dead staining results of MCF‐7 tumor cells after different treatments. The green and red fluorescence indicates the live and dead cells, respectively.

Tumor microenvironments (TMEs) are frequently characterized by acidic conditions, with pH levels ranging from 5.8 to 7.2, attributed to the abnormal metabolic activity within tumor cells.^[^ ^22^ ^]^ Given this context, the grouping C‐NRs and S‐MR@PSS@DOX particles is anticipated to execute the elimination of uncharted tumor cells through the precise collaborative mapping‐guided drug delivery. To implement this concept, MCF‐7 cells cultured on a coverslip were placed in the right reservoir of the microfluidic chip as a simulated tumor target, and the C‐NRs and S‐MR@PSS@DOX particles were navigated to collectively traverse the narrow canal in a leader‐follower‐like formation to perform precise tumor‐cell elimination (**Figure** **6**A). To replicate acidic TMEs, a pH 5.0 gel bead was placed alongside MCF‐7 cells on the coverslip, generating a localized acidic pH environment around the simulated tumor target (Figure 6B). To enhance the diagnostic capabilities of C‐NRs, the crosslinking degree of the poly(AA‐co‐HEA) hydrogel shell of C‐NRs was elaborately optimized from 2.4% to 2.3%, enabling them to manifest a noticeable structural color transition from red to yellow when detecting a subtle pH shift from the normal tissue pH of 7.4 to the tumor intercellular pH of 6.5 (Figure S15, Supporting Information).

When driven by the **H**_r_(*t*), the swarm successfully traversed the narrow canal with the C‐NRs taking the lead and approached the simulated tumor target placed in the right reservoir (0 – 94 s in Figure 6C; Video S14, Supporting Information). As the swarm continued its collective motion, the sensing C‐NRs gradually changed their structural color from red to yellow‐green, signifying the detection of the simulated tumor target (94 – 158 s in Figure 6C). In response to this diagnostic signal visualized by the structural color changes, the swarm was directed to move forward and drop the S‐MR@PSS@DOX particles onto the targeted MCF‐7 cells utilizing their selective self‐adhesion in the acidic environments (158 – 207 s in Figure 6C). Due to the high cytotoxicity of S‐MR@PSS@DOX particles, 96.5% of MCF‐7 cells were killed within 4 h (Figure 6D), as confirmed by fluorescence microscopic images of dead (red) and surviving (green) MCF‐7 cells (Figure 6E), revealing the outstanding therapeutic efficacy of the swarm. In contrast, no significant cell death was observed when the MCF‐7 cells were exposed to S‐MR@PSS particles (cell death rate < 7.3%) and the C‐NR/S‐MR@PSS mixture (cell death rate = 1.85%) (Figure 6D,E). These findings, along with dose‐dependent cytotoxicity and hemolytic assays (Figure S16, Supporting Information), indicate that the S‐MR@PSS particles are highly biocompatible, and the observed high cytotoxicity of S‐MR@PSS@DOX particles mainly originates from the loaded DOX. Therefore, the proposed heterogeneous swarm has great potential in precise tumor treatment.

## 3 Conclusion

In summary, we have demonstrated a general strategy for organizing disparate magnetic MNRs with significant differences in size, shape, and properties into cohesive microswarms with tunable heterogeneity and hierarchy, and explored their proof‐of‐concept collaborative serialized‐tasking capability in precise sensing/mapping‐guided drug delivery toward unknown targets. After systematically investigating their respective dependence of collective *U* on (*H*_0_, *f*) parameter sets of the external rotating **H**_r_(*t*), synchronization and desynchronization conditions of disparate magnetic MNRs (e.g., C‐NRs and S‐MRs) are easily determined. At the synchronization (*H*_0_, *f*) parameter sets, the disparate magnetic MNRs can reach motion synchronization in a mixed system, and, utilizing local attractive conspecific and interspecific hydrodynamic interactions, further form into a stable heterogeneous microswarm with an egalitarian group structure. By slightly desynchronizing the disparate MNRs by adjusting (*H*_0_, *f*) parameter sets, they undergo a process resembling “phase segregation” within the group, and leader‐follower‐like hierarchy could emerge in the heterogeneous microswarm. With this high controllability, the group configurations of the heterogeneous swarm can be adjusted from egalitarian to C‐NR‐leading or S‐MR‐leading configurations on the fly in dynamic manners, even when transversing an intricate microchannel with multiple sharp turns. The proposed strategy has been confirmed to be of great generality, as it can be used to construct heterogeneous swarms with tunable heterogeneity, including varying levels of heterogeneity (e.g., size differences) and diverse heterospecific pairings. By equipping them with the designated separate sensing and drug‐carrying functionality, the C‐NRs and S‐MRs organized in a leader‐follower‐like group configuration can execute precise drug delivery targeting uncharted targets in a collaborative sensing‐navigating‐cargo dropping sequence, and show great potential in precise tumor treatment. Our results highlight the importance of considering attribute differences and hierarchical organizations in the design of swarming biomedical micro/nanorobots, and may promote the development of intelligent micro/nanorobot systems with easy expandability, advanced swarm intelligence, complex cooperative multifunctionality, and high task‐accomplishing efficiency.

## 4 Experimental Section

### Materials

Sodium chloride (NaCl), cuprous bromide (CuBr), absolute ethanol, glycerol, acrylic acid (AA), 2‐hydroxyethyl acrylate (HEA), ethylene glycol dimethacrylate (EGDMA), 2‐hydroxy‐2‐methylpropiophenone (HMPP), ethylene glycol (EG), 2, 2‐bipyridyl (bpy), 2‐Bromo‐2‐methylpropionic acid (BMPA), 2‐Morpholinoethanesulphonic acid (MES), 1‐(3‐dimethylaminopropyl)−3‐ethylcarbodiimide hydrochloride (EDC), N‐hydroxysuccinimide (NHS), sodium styrene sulfonate (SSNa) purchased from Aladdin. Agar, Doxorubicin (DOX) hydrochloride, and Dulbecco's modified eagle medium (DMEM) were purchased from Aladdin. Penicillin/streptomycin (double antibody) from Capricorn. Fetal bovine serum (FBS) was purchased from GIBCO, Invitrogen Corp, USA. Calcin‐AM, propyridine iodide (PI), and cell counting kit‐8 (CCK‐8) were purchased from Beyotime Biotech Inc, China. MCF‐7 cells and endothelial cells were purchased from the Henan Engineering Research Center of Industrial Microbiology, China. The Ethylene Diamine Tetraacetic Acid (EDTA) anticoagulant rabbit blood sample was purchased from Wuhan Chundu Biotechnology Co., Ltd, China. None of the chemicals were further purified. 0.3 µm and 3 µm carboxyl‐modified and 3 µm amino‐modified magnetic beads (MBs) were purchased from Suzhou Nanomicro Technology Co Ltd, China. 1, 5, and 8 µm carboxyl‐modified MBs were purchased from BioMag Scientific Inc, China.

### Preparation of C‐NRs

The C‐NRs were prepared using the preparation methods previously reported.^[^ ^9^, ^18^ ^]^ Briefly, a precursor solution was prepared at first by mixing 0.75 mg mL^−1^ Fe_3_O_4_@PVP NPs,^[^ ^23^ ^]^ 300 mm AA, 127 mm HEA monomers, 13 mm crosslinker EGDMA, and 5 mm photoinitiator HMPP in 0.6 mL EG/water (5:1 in volume) solution. Then, the precursor solution was placed in a static magnetic field (*H*, 500 Gs) for 30 s. After being irradiated by UV light for 5 min and rinsed with the ethanol twice, the Fe_3_O_4_@poly(AA‐co‐HEA) C‐NRs were obtained, and then transferred into distilled water for later experiments. To enable the C‐NRs to manifest a noticeable structural color transition from red to yellow when detecting a subtle pH shift from the normal tissue pH of 7.4 to the tumor intercellular pH of 6.5, the crosslinking degree of the poly(AA‐co‐HEA) hydrogel shell of C‐NRs was elaborately optimized from 2.4% to 2.3%.

### Preparation of S‐MR@PSS Particles

At first, 1.67 g of BMPA, along with 1 mL of 200 mm MES aqueous solution, 1 mL of 1000 mm NaCl aqueous solution, 2 mL of 100 mm EDC aqueous solution, and 0.2 mL of 100 mm NHS aqueous solution, were mixed using a micro‐vortex mixer (Conian Biotech, TS100, China), maintained at room temperature and a stirring rate of 700 rpm for a duration of 30 min. Subsequently, 15 mg of amino‐modified MBs with a size of 3 µm was washed three times with ethanol and deionized water, respectively. Afterward, these beads were resuspended in 1.5 mL of deionized water to form a water suspension. The water suspension was then added into the activated solution and allowed to stir continuously for a period of 24 h. The product was washed 5 times with ethanol and deionized water, respectively, and dispersed in 2 mL of deionized water to obtain the intermediate product. In the final phase, a solution was prepared by dissolving 300 mg of SSNa, 10 mg of bpy, and 5 mg of CuBr in 8 mL of deionized water. To this solution, 2 mL of the intermediate product was added. The system was then degassed for three pump cycles, and maintained at 70 °C for 9 h under an N_2_ atmosphere. The reaction was quenched by exposure to the air, and the obtained S‐MRs@PSS particles were immediately separated from the solution by magnetic separation and thoroughly washed with ethanol and deionized water.

### Characterization

Optical bright‐field, dark‐field, and fluorescence microscopic images were captured with an inverted optical microscope (Leica DMI 3000 M, Germany). SEM and TEM images were acquired by field emission scanning electron microscopy (Hitachi S‐4800, 10 kV, Japan) and high‐resolution transmission electron microscopy (JEOL JEM‐2100F, 200 kV, Japan), respectively. The FT‐IR spectrum was obtained by the Fourier transform infrared spectrometer (Thermo, Nicolet 6700, USA). Hysteresis loops were obtained by the vibrating sample magnetometer (Lake Shore, 7404, USA). The Zeta potential and hydrodynamic diameter of the particles were measured by a zeta potential and particle size analyzer (Brookhaven, NanoBrook 90Plus Zeta, USA).

### Magnetic Propulsion

C‐NRs with a concentration of 2 mg mL^−1^ and S‐MRs (3 µm MBs) at a concentration of 0.75 mg mL^−1^ were mixed at different mass ratios utilizing a micro‐vortex mixer, and stored for further experiments. The magnetic propulsion experiments were conducted within a custom magnetic field system comprising a current source (TA‐309 power amplifier, China), a signal source (NI USB‐6343, USA), and a 3‐axis Helmholtz coil. A 20 µL evenly mixed water suspension of C‐NRs and S‐MRs with different mass ratios was dripped onto a glass substrate or into a glass chamber and collected near the substrate using a permanent magnet (3000Gs). Then, the substrate or chamber was transferred to the coil mounted on an optical microscope, and, by applying a rotating **H**_r_(*t*) with different directions, *H*_0_ and *f*, the C‐NRs and S‐MRs were propelled and organized into microswarms with desired group configurations for navigation or to perform tasks. The collective motion of the swarm was observed and recorded from the top and side using an inverted optical microscope (Leica DMI3000B, Germany) and a stereomicroscope (Olympus SZX16, Japan), respectively. In addition, the swarming motions of the C‐NRs and S‐MRs in water containing passive polystyrene (PS) tracers (2 µm, 0.6 mg mL^−1^), in aqueous glycerol media with different viscosities (1.3 and 3.0 cP), and on different substrates (glass, endothelial cell‐seeded glass, and plastic) were also investigated following the similar procedures, respectively.

### Numerical Simulations

The pH‐distribution simulations were performed using the diffusion, electrostatics, and laminar flow modules of COMSOL Multiphysics software. The simulation model was built using a cylindric reservoir (radius *r* = 6 mm, height *h* = 0.5 mm) connected to a narrow canal (width *a* = 1.5 mm, *h* =  0.5 mm, and length *l* = 8 mm). The reservoir and canal were filled with water (pH 7.4). A fluidic flow with a velocity of 87 µm s^−1^ was injected from the left narrow canal into the cylindric reservoir, simulating the flow of swarming micro/nanorobots. The diffusion constant of H^+^ and OH^−^ was set to be 9.3 × 10^−9^ and 5.2 × 10^−9^ m^2^ s^−1^. The pH distribution in the cell experiment was obtained by simulating the diffusion of H^+^ and OH^−^ from a 2 mm sodium alginate bead (surface pH 5.0) at the position 8.2 mm from the far‐right reservoir edge in the middle (Figure 6B). The numerical simulation of the flow field around a wheel‐like S‐MR swarm and a C‐NR was performed using the laminar flow module of COMSOL Multiphysics software. The wheel‐like S‐MR swarm and the C‐NR were placed in a cuboid reservoir (*l* = 170 µm, *a*  = 50 µm, and *h* = 50 µm) filled with water. The rotation frequency of the wheel‐like S‐MR swarm and the C‐NR was adjusted from 1 to 13 Hz, and the distance between them was adjusted from 5 to 20 µm. The interaction forces between the wheel‐like S‐MR swarm and the C‐NR and that between the C‐NR and glass substrate were obtained by integrating the total stress (N m^−2^ in unit) in the *x*‐axis and *z*‐axis directions at each point over their edges, respectively.

### Drug Loading Experiment

A DOX solution was prepared by dissolving 2 mg of DOX in 2 mL of water. Subsequently, 1 mL of S‐MR@PSS (5 mg mL^−1^) particles was introduced into the aqueous DOX solution, and the mixture was subjected to agitation within a micro‐vortex mixer at a stirring rate of 700 rpm for a duration of 12 h. Finally, the obtained S‐MR@PSS@DOX particles were magnetically separated, washed with deionized water and phosphate buffer solutions (PBS) respectively for 3 times and then stored in PBS for later use.

### Collaborative Precise Drug Delivery

The collaborative precise drug delivery was conducted within a microfluidic chip with two open reservoirs interconnected by a narrow canal. First, the microfluidic chip was filled with 1 mL PBS buffer, and then the aqueous suspension of well‐mixed C‐NRs (2 mg mL^−1^) and S‐MRs@PSS@DOX particles suspended in water (0.75 mg mL^−1^) was dropped into a reservoir at one end of the microchannel (20 µL), and then the robots were collected near the substrate using a permanent magnet. Then, test targets (agar gels with different pH values) were placed at the other reservoir of the microchannel. An **H**_r_(*t*) with a (*H*_0_, *f*) parameter set of (16 mT, 1 Hz) was applied to navigate the C‐NR‐S‐MR@PSS@DOX swarm to cross the canal, detect the low‐pH target, cover the target, and complete the precise drug delivery. The experiments were observed and recorded under dark field microscopy using an inverted optical microscope.

### Targeted Tumor‐Cell Elimination

All MCF‐7 cells were cultured in an incubator maintained at 37 °C and 5% CO_2_. Prior to use, all materials underwent sterilization by exposure to ultraviolet light. First, MCF‐7 cells with a density of 60 000 cm^−2^ were inoculated on a 9 mm coverslip for 24 h. Subsequently, the coverslip was transferred to a reservoir of the microfluidic chip. To replicate acidic TMEs, a sodium alginate bead (≈ 2 mm in size) loaded with pH 5.0 PBS buffer was placed on the coverslip with MCF‐7 cells. Immediately afterward, a 30 µL suspension of mixed C‐NRs (3.33 mg mL^−1^) and S‐MR@PSS@DOX particles (0.42 mg mL^−1^) was introduced into the other reservoir of the microchannel and collected using a permanent magnet. An **H**_r_(*t*) with a (*H*_0_, *f*) parameter set of (16 mT, 1 Hz) was applied to activate the C‐NR‐S‐MR@PSS@DOX swarm to cross the canal, detect the low‐pH target, cover the target, and complete the precise drug delivery. The viability of MCF‐7 cells was evaluated by live/dead staining. Imaging analysis was performed using the inverted fluorescence microscope after staining with 4.5 µm PI (red for dead cells) and 2 µm Calcein‐AM (green for living cells) at 37 °C for 30 min. The cell viability of MCF‐7 cells without and with exposure to equal amounts of S‐MR@PSS particles and C‐NRs was tested under the same conditions.

### Cytotoxicity Study

MCF‐7 cells were inoculated into 96‐well plates at a seeding density of 8000 cells per well and subsequently cultivated in 100 µL of DMEM supplemented with a 1% penicillin/streptomycin mixture and 10% FBS for a duration of 24 h. Following this incubation period, the culture medium was aspirated, and MCF‐7 cells were co‐cultured with 100 µL of fresh medium, each containing varying concentrations (ranging from 0 to 0.125 mg mL^−1^) of S‐MR@PSS particles for an additional period of 24 h. Subsequently, each well was incubated in complete DMEM containing 10% CCK‐8 by volume at 37 °C for 2 h. The particles were then concentrated at the bottom with a permanent magnet. The supernatant was transferred to a new 96‐well plate, and the absorbance of the supernatant was measured at 450 nm using a microplate reader (PerkinElmer, HH3400, USA) to calculate cell viability.

### Hemolysis Assay

Initially, 2 mL of rabbit blood samples anticoagulated with EDTA were added to 5 mL of PBS, following which the red blood cells (RBCs) were separated from the serum through centrifugation at 1500 rpm for a duration of 5 min. The resultant purified blood samples were subjected to three wash cycles with 5 mL of PBS solution and were subsequently diluted to a concentration of 1/10 relative to the initial volume, using PBS solution. Subsequently, 0.5 mL of the diluted RBC suspension was mixed with the following solutions: (a) 2.5 mL of PBS, serving as a negative control, (b) 2.5 mL of deionized water, functioning as a positive control, and (c) a suspension of S‐MR@PSS with varying concentrations (ranging from 0.0025 to 0.125 mg mL^−1^). Each group was thoroughly mixed and left at room temperature for a duration of 3 h. Finally, the absorbance of the supernatant was measured at 540 nm using the microplate reader.

### Statistical Analysis

All data were presented as mean ± standard deviation (SD) from at least three independent experiments. The sample size and result obtention have been added to the corresponding figure captions. All images were subsequently subjected to analysis using Adobe Premiere Pro 2021 and ImageJ software.

## Conflict of Interest

The authors declare no conflict of interest.

## Supporting information

Supporting Information

Supplemental Video 1

Supplemental Video 2

Supplemental Video 3

Supplemental Video 4

Supplemental Video 5

Supplemental Video 6

Supplemental Video 7

Supplemental Video 8

Supplemental Video 9

Supplemental Video 10

Supplemental Video 11

Supplemental Video 12

Supplemental Video 13

Supplemental Video 14

## Data Availability

The data that support the findings of this study are available in the supplementary material of this article.
