# Supplementary material for: Harnessing Disparities in Magnetic Microswarms: From Construction to Collaborative Tasks
Source: Adv Sci (Weinh). 2024 Jun 13;11(30):2401711. doi: 10.1002/advs.202401711 (PMC11321641; doi:10.1002/advs.202401711)
Supplement: Supplementary file 1 — Supporting Information [file ADVS-11-2401711-s013.docx]

Supporting Information

Harnessing Disparities in Magnetic Microswarms: From Construction to Collaborative Tasks

Chuan Cao, Fangzhi Mou*, Manyi Yang, Shuming Zhang, Di Zhang, Luolin Li, Tong Lan, Dunyi Xiao, Wei Luo, Huiru Ma*, and Jianguo Guan*

Supporting Figures


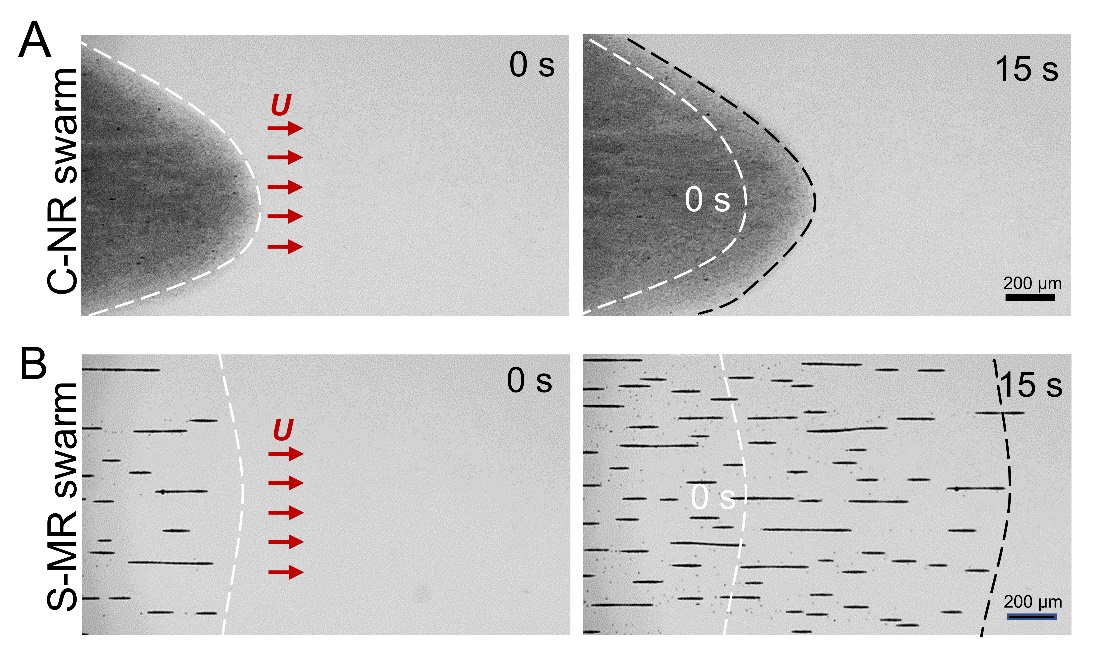


**Figure S1.** Time-lapse microscopic images depicting collective motions of homogeneous swarms of (A) C-NRs and (B) S-MRs under a rotating **H**_r_(*t*) with an *H*_0_ of 16 mT and an *f* of 7 Hz.


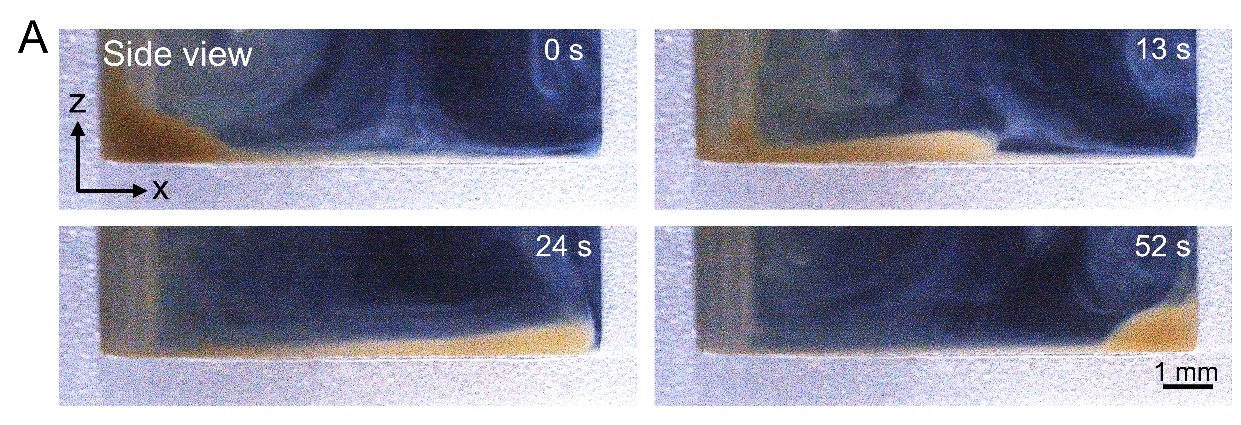


**Figure S2.** Time-lapse microscopic images in a side view (*x-z* plane) depicting the shape of the entire heterogeneous swarm as it moves rightward.


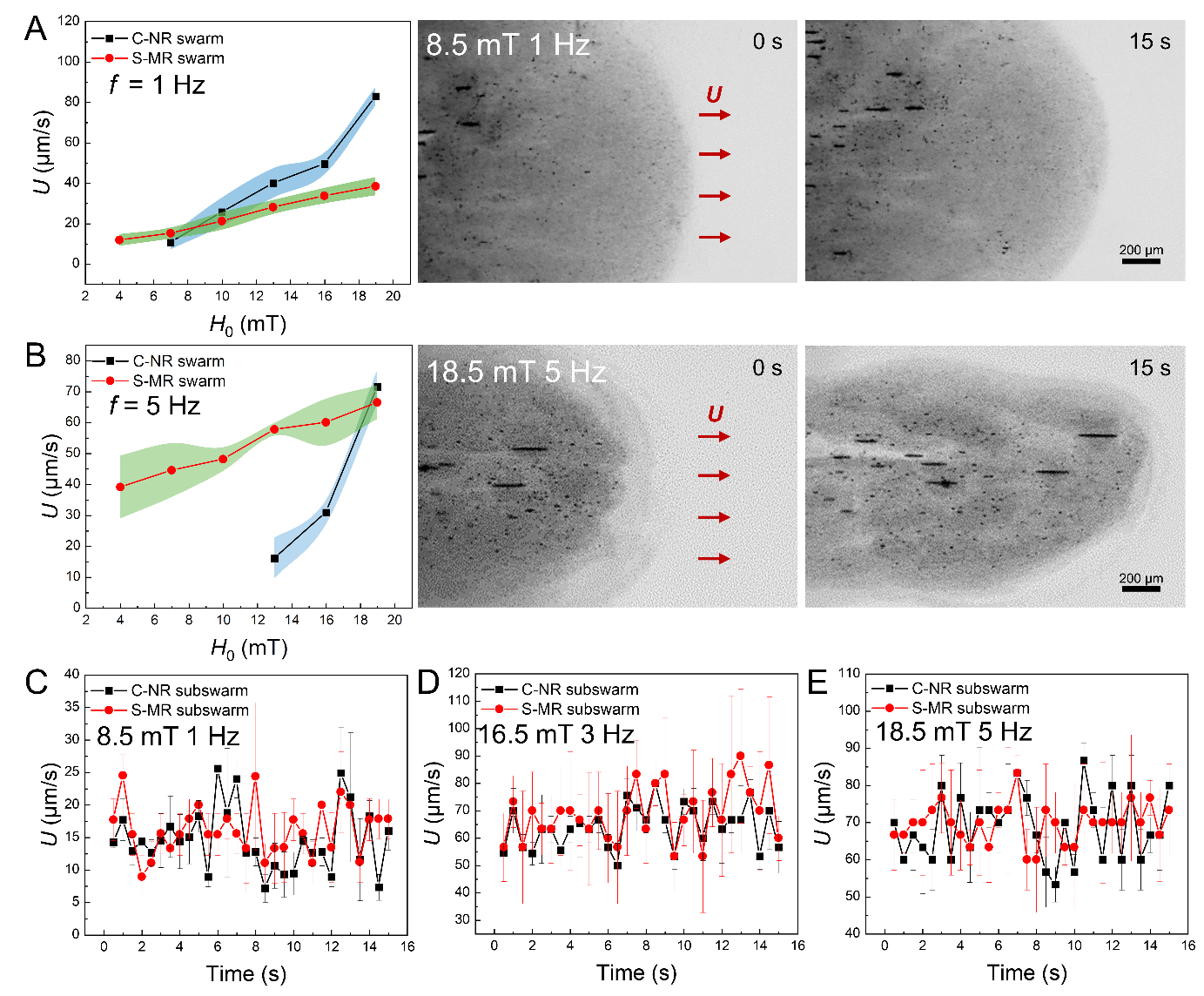


**Figure S3.** (A, B) Collective velocity (*U*) of swarming C-NRs and swarming S-MRs as a function of *H*_0_ when *f* is 1 (A) and 5 (B) Hz (left panels), and time-lapse microscopic images (right panels) depicting the collective motion of the corresponding heterogeneous swarms driven at the determined synchronization *H*_0_ parameter. (Sample size, *n* = 5, results are shown as mean ± SD). (C-E) Instantaneous speed of C-NR subswarm and S-MR subswarm at the synchronization (*H*_0_, *f*) parameter sets of (8.5 mT, 1 Hz) (C), (16.5 mT, 3 Hz) (D) and (18.5 mT, 5 Hz) (E), respectively. (*n* = 5, results are shown as mean ± SD).


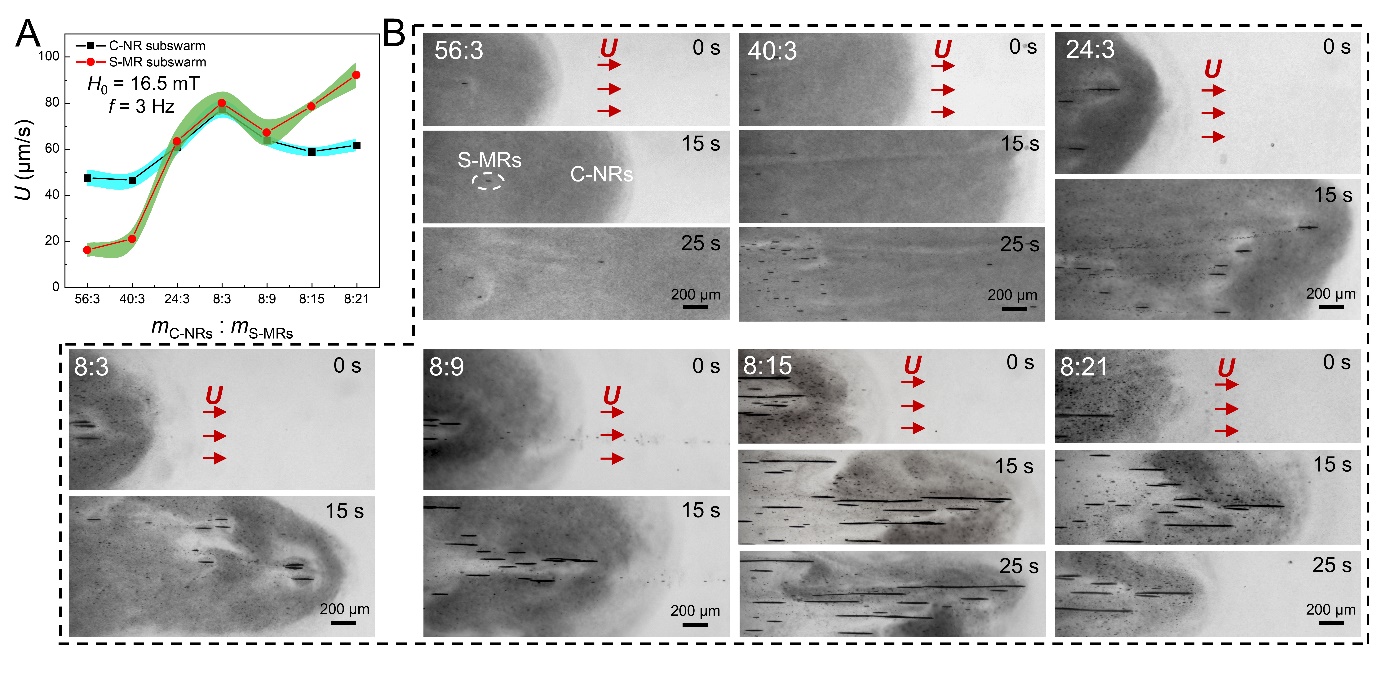


**Figure S4.** (A) Collective velocity *U* of C-NRs subswarm and S-MRs subswarm as a function of mass ratio (*m*_C-NRs_ : *m*_S-MRs_). (*n* = 5, results are shown as mean ± SD). (B) Time-lapse microscopic images of the heterogeneous swarms with different C-NR to S-MR ratios.


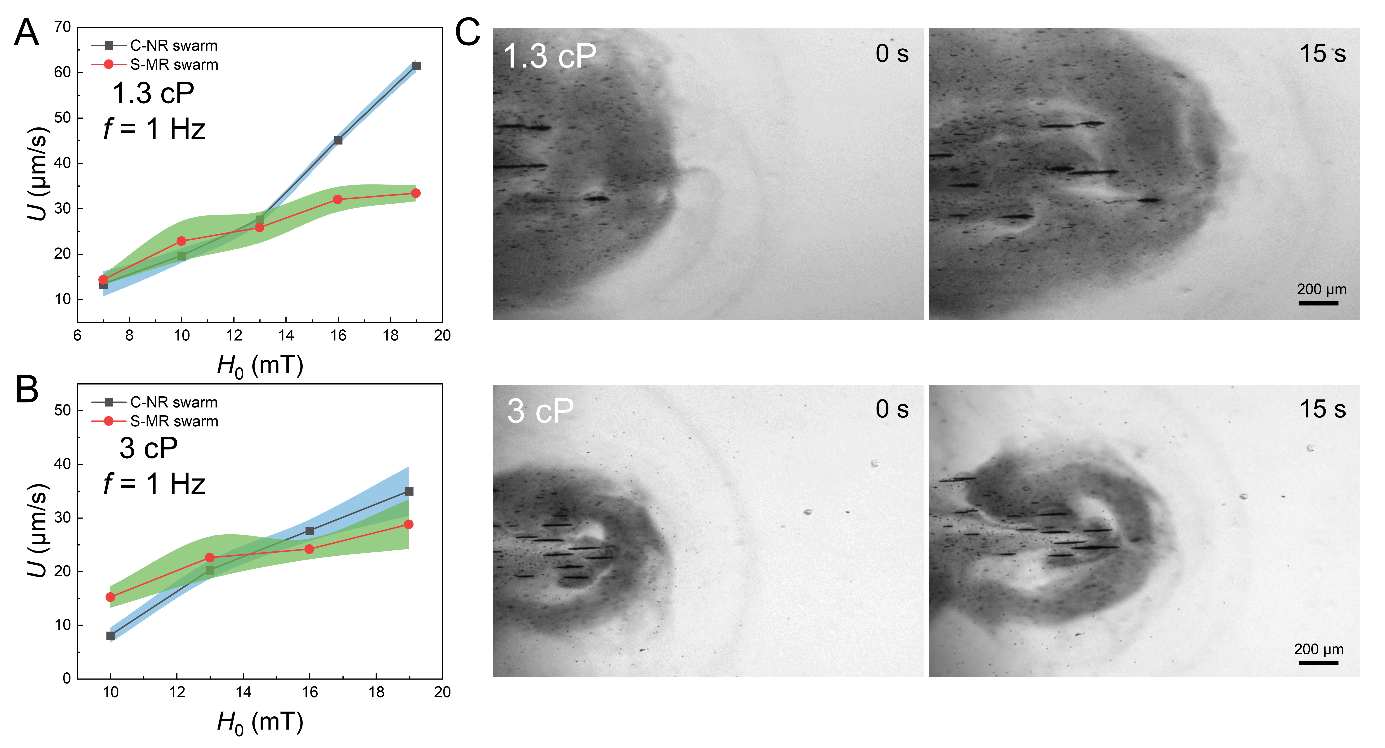


**Figure S5.** (A, B) Collective velocity *U* of C-NRs and S-MRs as a function of *H*_0_ in the liquid medium with a viscosity of 1.3 (A) and 3.0 cP (B) (left panels), respectively. (*n* = 5, results are shown as mean ± SD). (C) The corresponding time-lapse microscopic images depicting thier collective motion at the determined synchronization *H*_0_ parameter. The *f* is kept at 1 Hz.


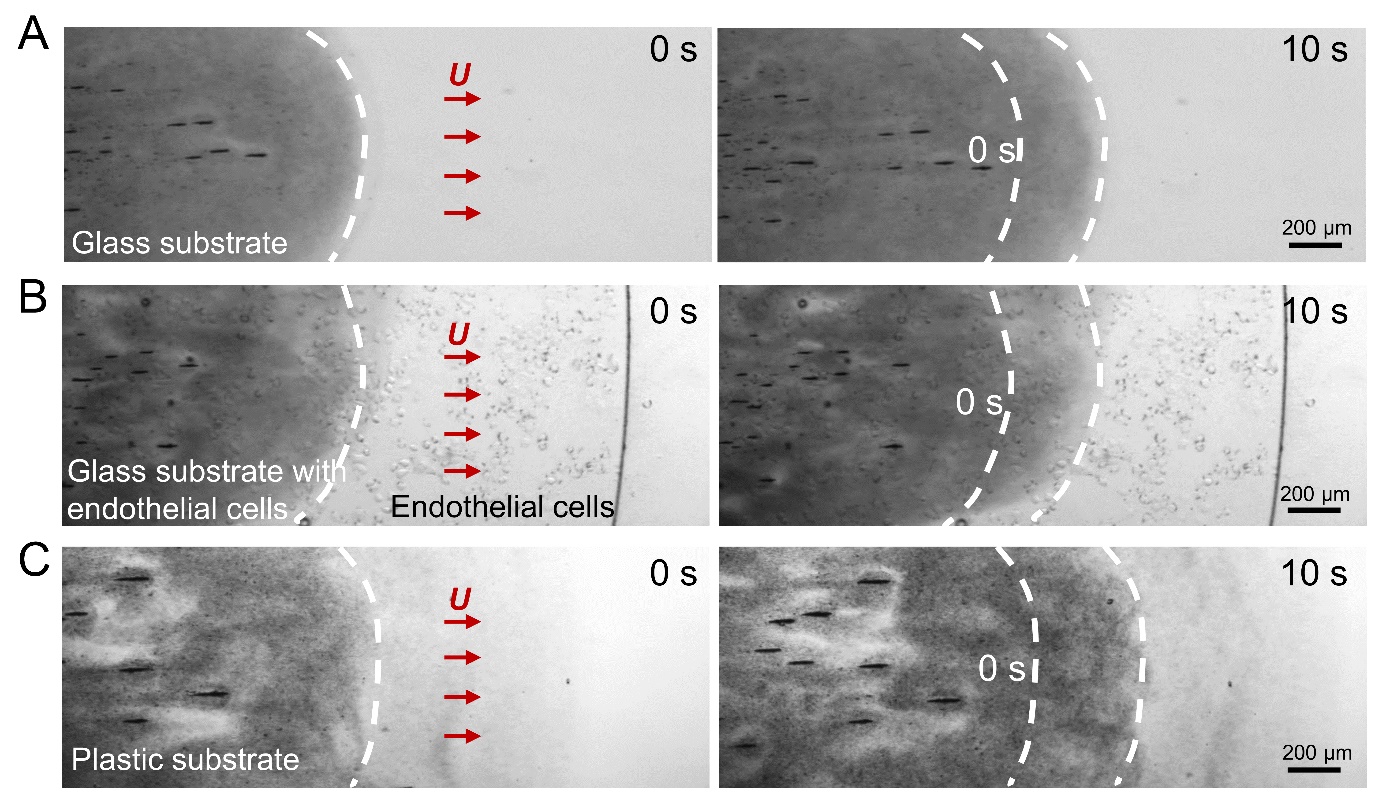


**Figure S6.** Time-lapse microscopic images depicting the collective motions of C-NRs and S-MRs on the (A) glass substrate, (B) endothelial cell-seeded glass substrate, and (C) plastic substrate when driven at a (*H*_0_, *f*) parameter set of (8.5 mT, 1 Hz).


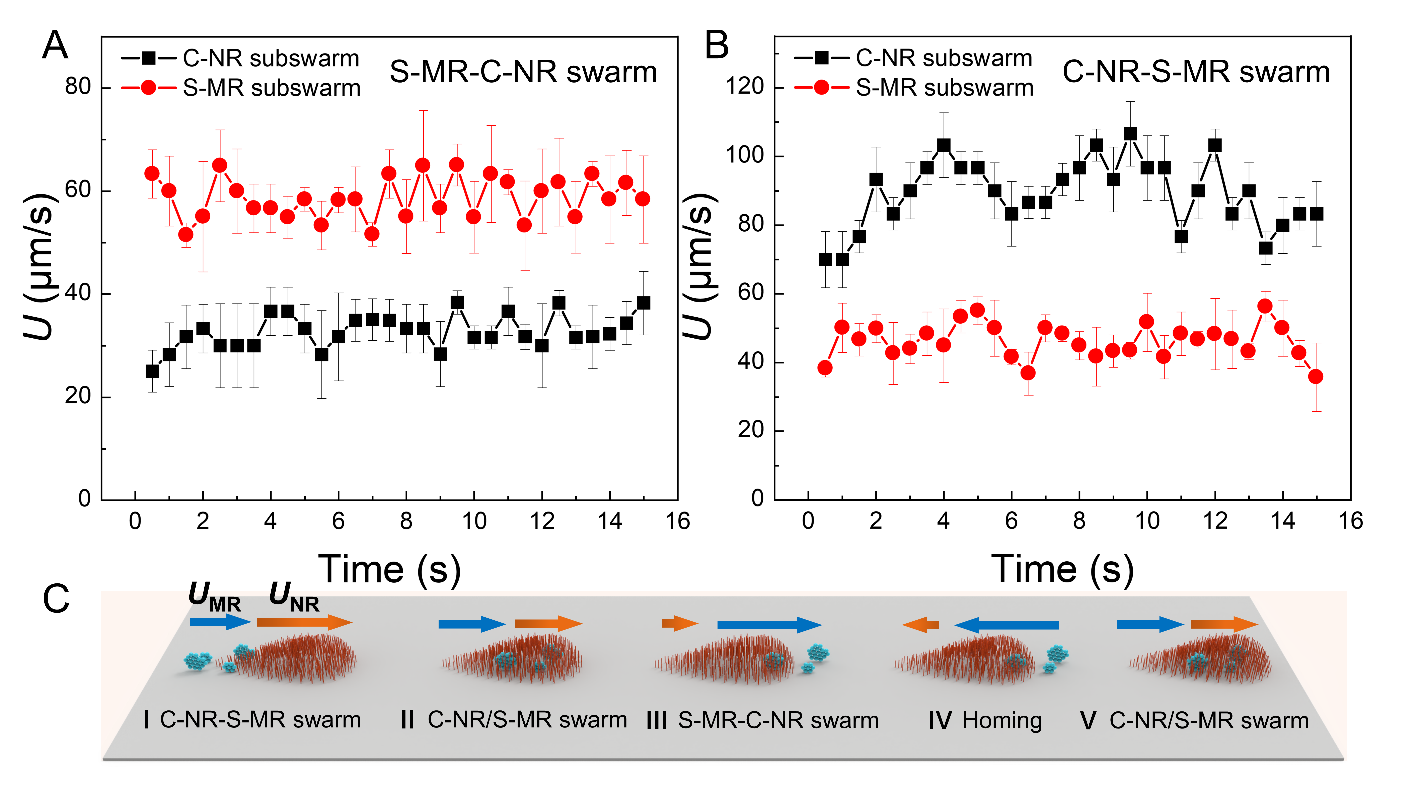


**Figure S7.** (A, B) Instantaneous *U* of C-NR subswarm and S-MR subswarm in the S-MR-C-NR (A) and C-NR-S-MR (B) hierarchical heterogeneous swarm, respectively. (*n* = 5, results are shown as mean ± SD). (C) Schematic illustration of dynamic group configuration transformations of a heterogeneous swarm in open space. *U*_NR_ and *U*_MR_ are the collective velocities of C-NRs and S-MRs, respectively.


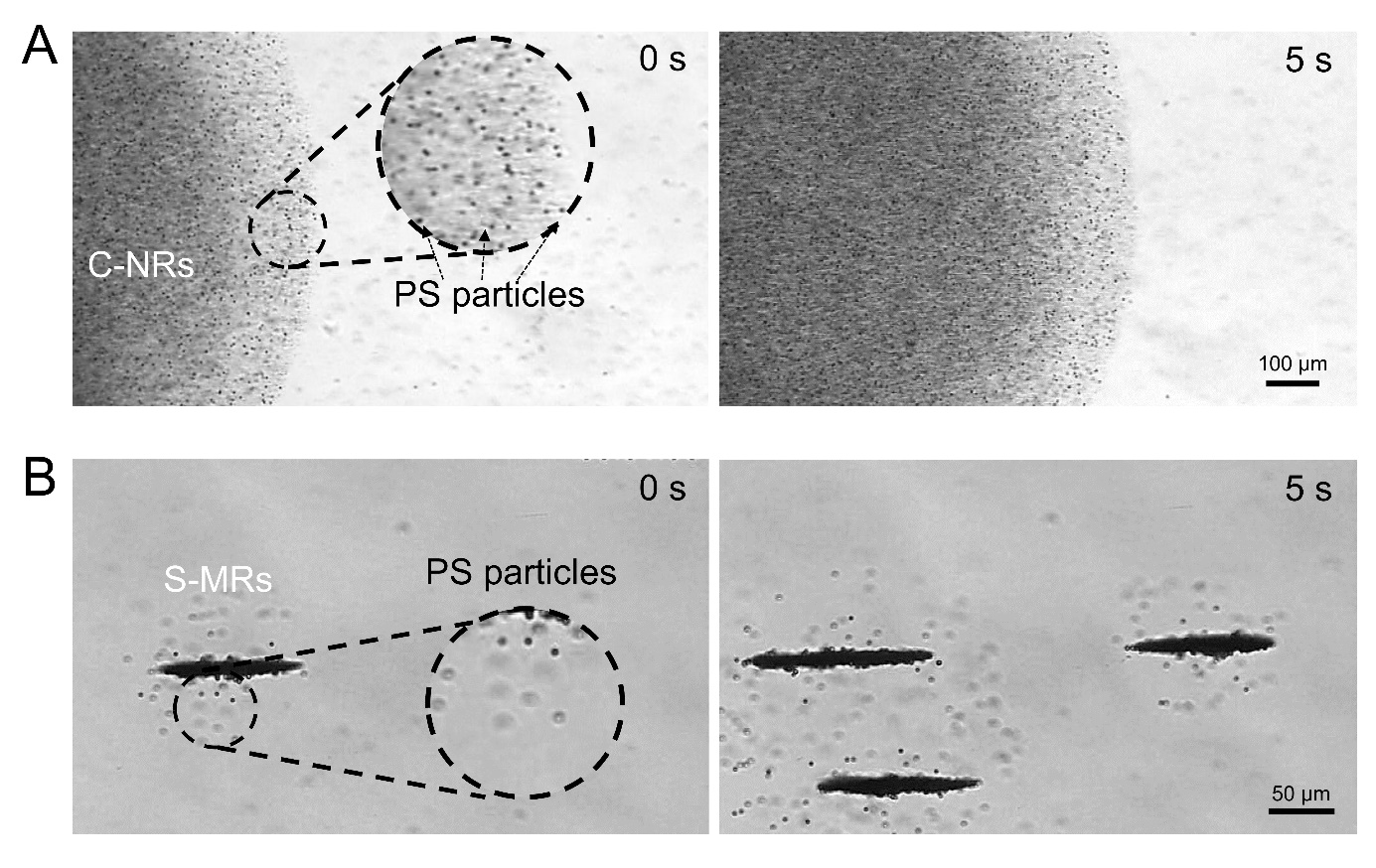


**Figure S8.** Time-lapse microscopic images depicting the collective motions of (A) C-NRs and (B) S-MRs when mixed with passive tracers of polystyrene (PS) microsphere (2 μm), respectively.


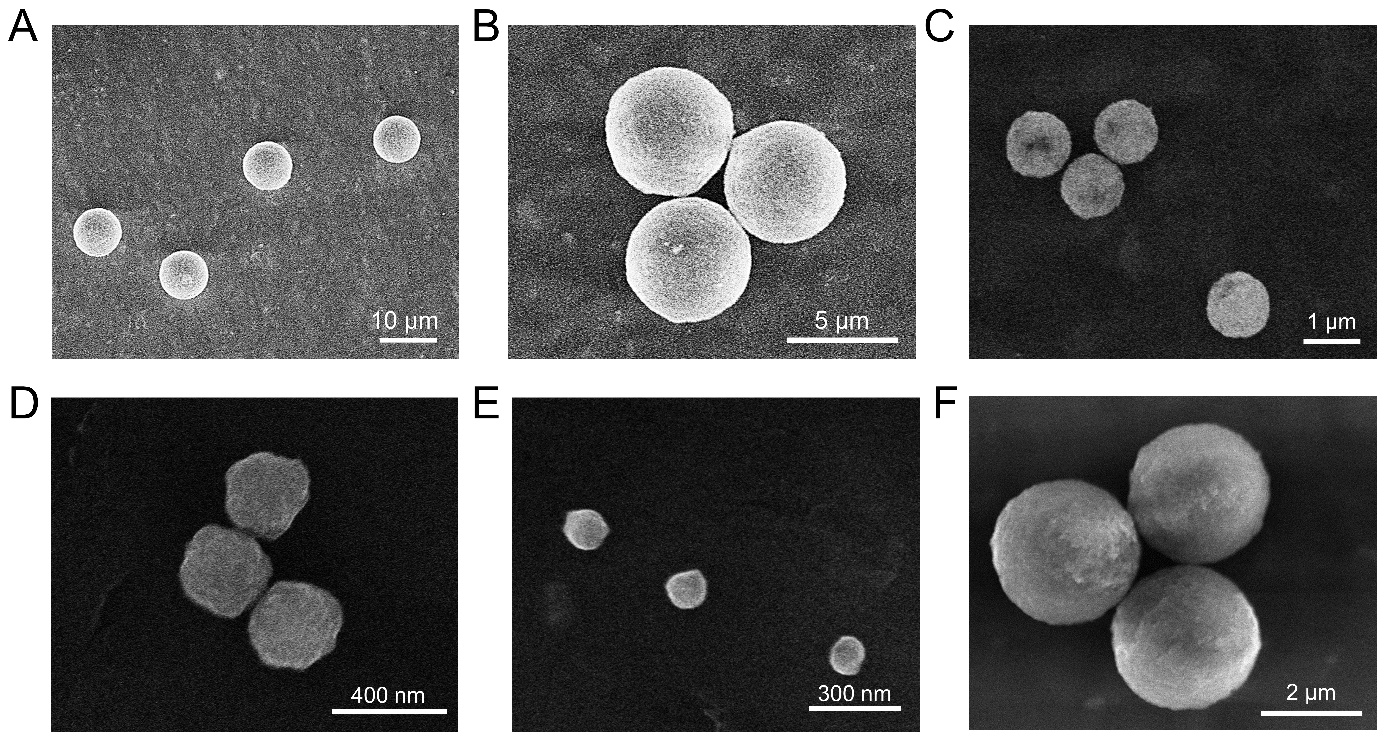


**Figure S9.** (A-E) SEM images of carboxyl-modified MBs with a size of 8 (A), 5 (B), 1 (C), 0.3 (D), and 0.15 μm (E), respectively. (F) The SEM image of 3 μm amino-modified MBs.


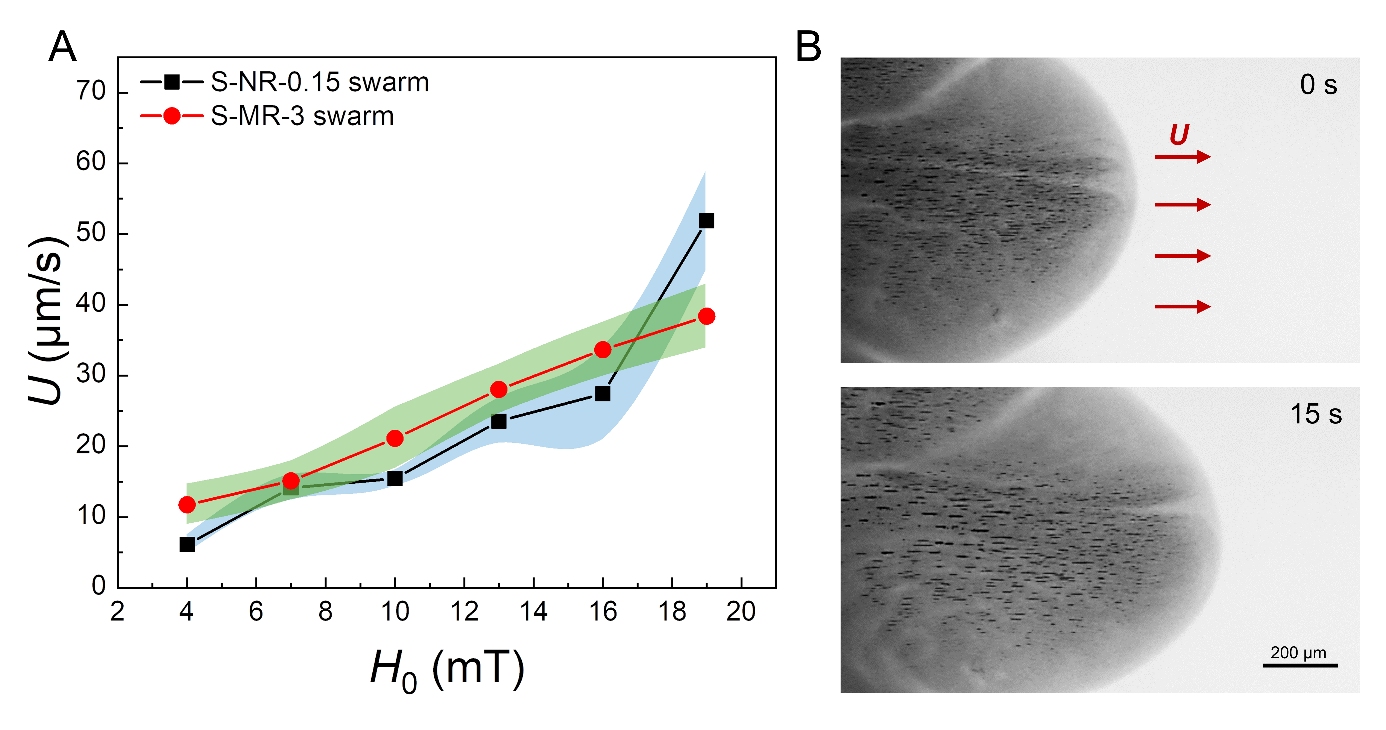


**Figure S10.** (A) Collective velocity *U* of S-MR-3 swarms and those swarms consisting of spherical magnetic nanoparticles (150 nm) (S-NR-0.15 swarms) as a function of *H*_0_. The *f* is kept at 1 Hz. (*n* = 5, results are shown as mean ± SD). (B) Time-lapse microscopic images depicting the collective motion of the corresponding heterogeneous S-MR-3/S-NR-0.15 swarms when driven at a (*H*_0_, *f*) parameter set of (7 mT, 1 Hz).


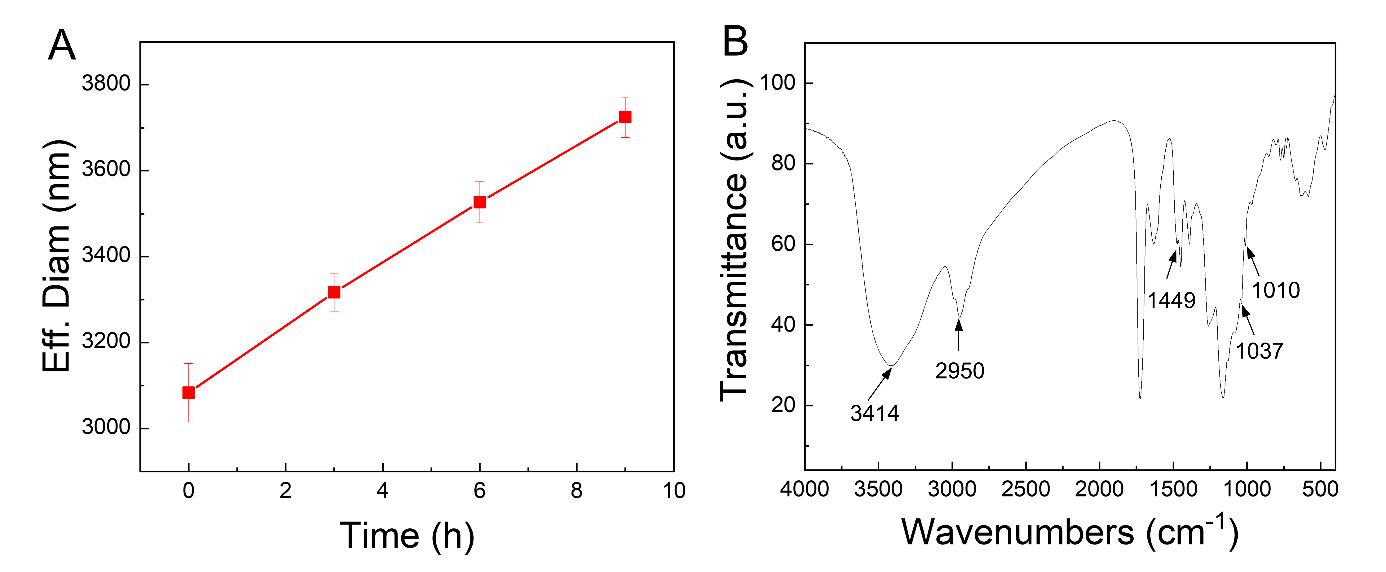


**Figure S11.** (A) The hydrodynamic diameter of S-MR@PSS particles obtained at different ATRP times. (*n* = 3, results are shown as mean ± SD). (B) The FT-IR spectrum of S-MR@PSS particles.


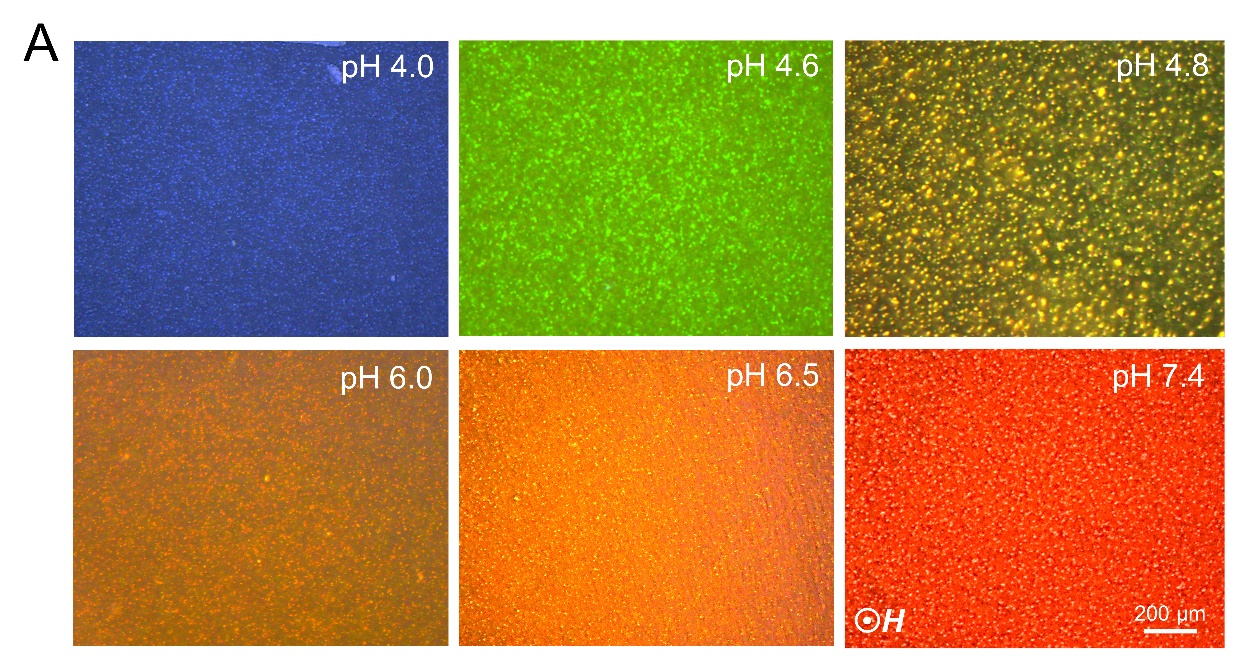


**Figure S12.** Dark-field optical microscopic images of C-NRs at different pH values.


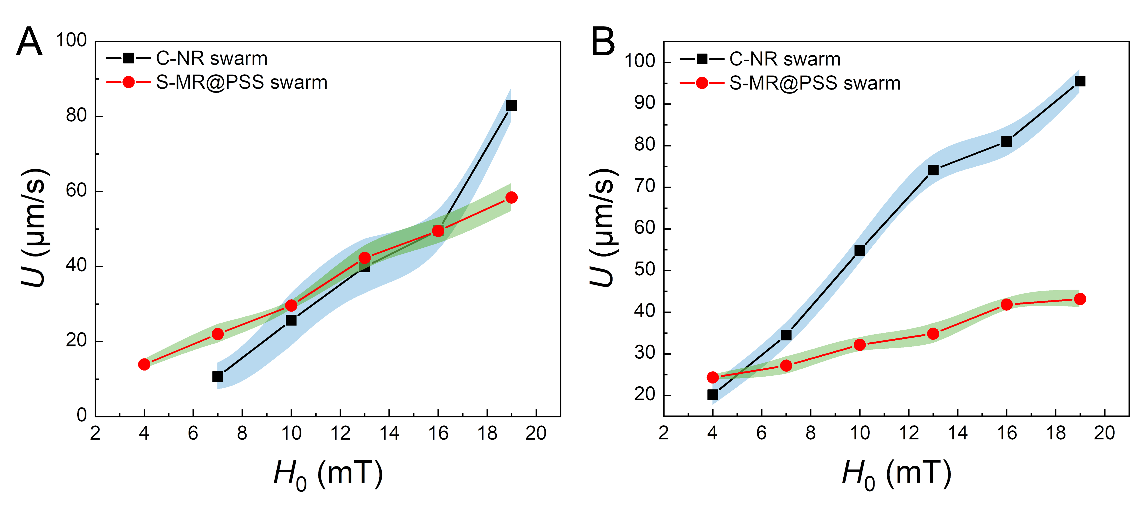


**Figure S13.** Collective velocity *U* of swarming C-NRs and swarming S-MR@PSS@DOX particles as a function of *H*_0_ in (A) open space and within the (B) confined microchannel. The *f* is kept at 1 Hz. (*n* = 5, results are shown as mean ± SD).


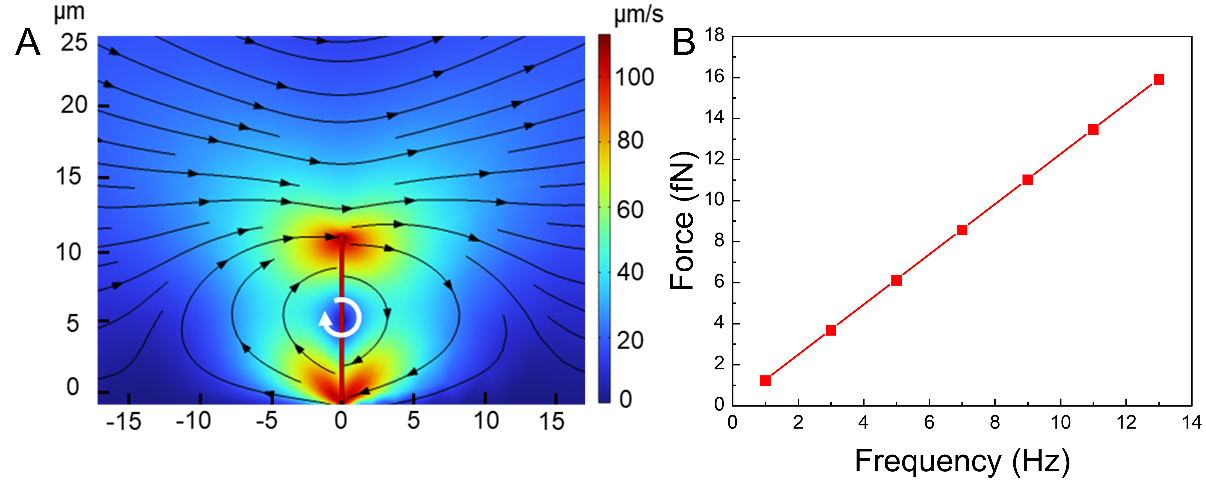


**Figure S14.** (A) Numerical simulation of the flow field around a C-NR at a rotating frequency *f* of 1 Hz. (B) The interaction force between the C-NR and substrate at different *f*, which is derived from simulation results in A.


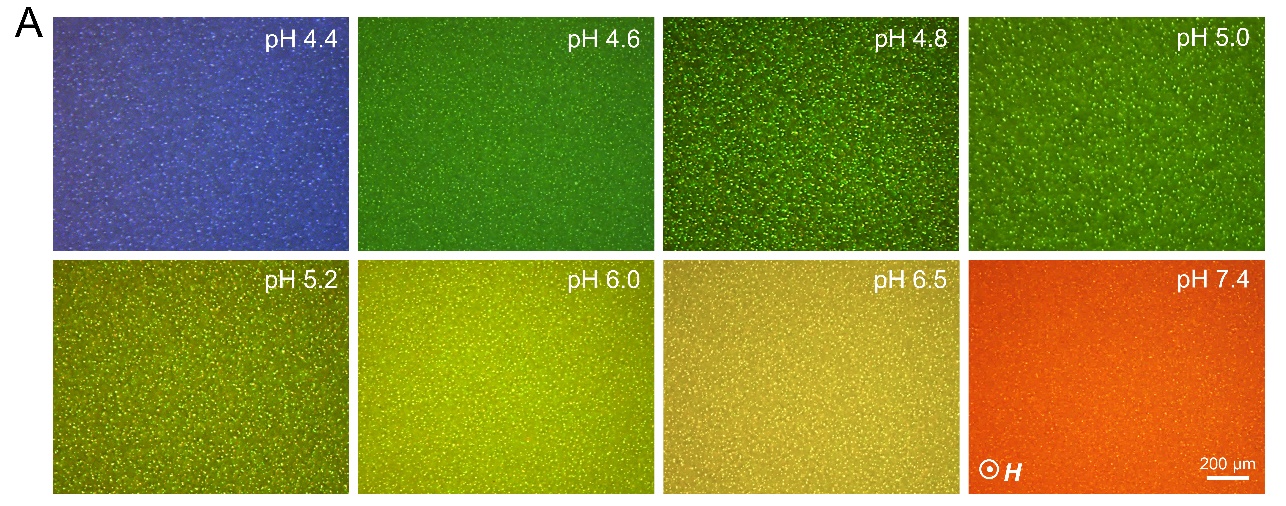


**Figure S15.** Dark-field optical microscopic images of C-NRs at different pH values after the crosslinking degree of the poly(AA-co-HEA) hydrogel shell of C-NRs was optimized to 2.3%.


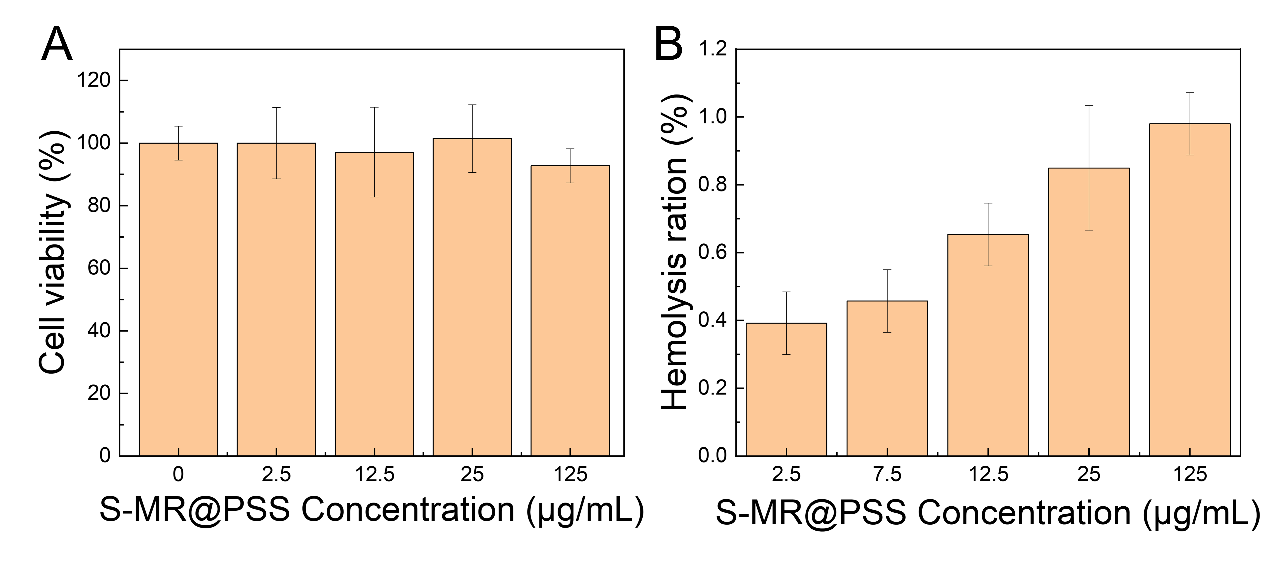


**Figure S16.** (A) MCF-7 cells viability after co-incubation with S-MRs@PSS particles at different concentrations for 24 h. (*n* = 3, results are shown as mean ± SD). (B) Hemolysis rate of S-MRs@PSS particles at different concentrations. (*n* = 3, results are shown as mean ± SD).

Supporting Videos

**Video S1:** Collective motion of swarming C-NRs and S-MRs at a (*H*_0_, *f*) parameter set of (16 mT, 7 Hz).

**Video S2:** Collective motion of the heterogeneous swarm at a (*H*_0_, *f*) parameter set of (16.5 mT, 3 Hz) in a side view.

**Video S3:** Collective motions of C-NRs and S-MRs in the egalitarian heterogeneous swarms at different synchronization conditions.

**Video S4:** Collective motion of C-NRs and S-MRs mixed in different mass ratios.

**Video S5:** Collective motion of C-NRs and S-MRs in the media with different viscosities.

**Video S6:** Collective motions of C-NRs and S-MRs on different substrates.

**Video S7:** Collective motions of C-NRs and S-MRs in the hierarchical S-MR-C-NR and C-NR-S-MR heterogeneous swarms.

**Video S8:** Dynamic group configuration transformations of the heterogeneous swarm.

**Video S9:** The navigation of a heterogeneous swarm in a microchannel with multiple sharp turns.

**Video S10:** Collective motions of C-NRs and S-MRs when mixed with passive PS tracers (2 μm), respectively.

**Video S11:** Collective motions of C-NR/S-MR-a heterogeneous swarms.

**Video S12:** Collaborative precise drug delivery by the C-NR-S-MR@PSS@DOX swarm when collectively moving from a reservoir (pH 7.4) toward an unknown low-pH target (a pH 4.4 agar gel).

**Video S13:** Collaborative precise drug delivery by the C-NR-S-MR@PSS@DOX swarm when collectively moving from a reservoir (pH 7.4) toward an unknown low-pH target (a pH 4.4 agar gel) with an interference (a pH 7.4 agar gel).

**Video S14:** Precise collaborative mapping-guided drug delivery of the C-NR-S-MR@PSS@DOX swarm toward MCF-7 tumor cells.
